# Circulating tumour DNA profiling reveals heterogeneity of EGFR inhibitor resistance mechanisms in lung cancer patients

**DOI:** 10.1038/ncomms11815

**Published:** 2016-06-10

**Authors:** Jacob J. Chabon, Andrew D. Simmons, Alexander F. Lovejoy, Mohammad S. Esfahani, Aaron M. Newman, Henry J. Haringsma, David M. Kurtz, Henning Stehr, Florian Scherer, Chris A. Karlovich, Thomas C. Harding, Kathleen A. Durkin, Gregory A. Otterson, W. Thomas Purcell, D. Ross Camidge, Jonathan W. Goldman, Lecia V. Sequist, Zofia Piotrowska, Heather A. Wakelee, Joel W. Neal, Ash A. Alizadeh, Maximilian Diehn

**Affiliations:** 1 Institute for Stem Cell Biology and Regenerative Medicine, Stanford University, Stanford, California 94305, USA; 2 Stanford Cancer Institute, Stanford University, Stanford, California 94305, USA; 3 Clovis Oncology, Inc., San Francisco, California 94158, USA; 4 Division of Oncology, Department of Medicine, Stanford University, Stanford, California 94305, USA; 5 Department of Bioengineering, Stanford University, Stanford, California 94305, USA; 6 Molecular Graphics and Computation Facility, College of Chemistry, University of California, Berkeley, California 94720, USA; 7 The Ohio State University, Columbus, Ohio 43210, USA; 8 Division of Medical Oncology, Department of Medicine, University of Colorado School of Medicine, Aurora, Colorado 80045, USA; 9 David Geffen School of Medicine, University of California, Los Angeles, Los Angeles, California 90095, USA; 10 Massachusetts General Hospital & Harvard Medical School, Boston, Massachusetts 02115, USA; 11 Division of Hematology, Department of Medicine, Stanford University, Stanford, California 94305, USA; 12 Department of Radiation Oncology, Stanford University, Stanford, California 94305, USA

## Abstract

Circulating tumour DNA (ctDNA) analysis facilitates studies of tumour heterogeneity. Here we employ CAPP-Seq ctDNA analysis to study resistance mechanisms in 43 non-small cell lung cancer (NSCLC) patients treated with the third-generation epidermal growth factor receptor (EGFR) inhibitor rociletinib. We observe multiple resistance mechanisms in 46% of patients after treatment with first-line inhibitors, indicating frequent intra-patient heterogeneity. Rociletinib resistance recurrently involves *MET*, *EGFR*, *PIK3CA*, *ERRB2*, *KRAS* and *RB1*. We describe a novel EGFR L798I mutation and find that EGFR C797S, which arises in ∼33% of patients after osimertinib treatment, occurs in <3% after rociletinib. Increased *MET* copy number is the most frequent rociletinib resistance mechanism in this cohort and patients with multiple pre-existing mechanisms (T790M and *MET*) experience inferior responses. Similarly, rociletinib-resistant xenografts develop *MET* amplification that can be overcome with the MET inhibitor crizotinib. These results underscore the importance of tumour heterogeneity in NSCLC and the utility of ctDNA-based resistance mechanism assessment.

## Abstract (web summary)

*EGFR*-mutant non-small cell lung cancer is routinely treated with EGFR inhibitors, although resistance inevitably develops. Here, the authors sequence circulating tumour DNA and show that resistance to the third-generation inhibitor rociletinib is heterogeneous and recurrently involves somatic alterations of *MET*, *EGFR*, *PIK3CA*, *ERRB2*, and *KRAS*.

## Graphical abstract

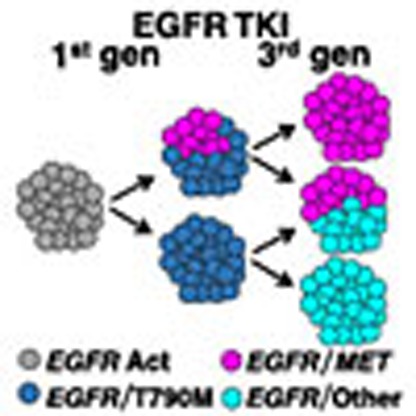

Activating mutations in epidermal growth factor receptor (EGFR) sensitize the majority of non-small cell lung cancer (NSCLC) tumours harbouring these lesions to EGFR tyrosine kinase inhibitors (TKIs)^1^,^2^,^3^. First-generation inhibitors such as erlotinib and gefitinib target the receptor via reversible binding of the tyrosine kinase domain, while second-generation TKIs such as afatinib covalently bind the target. Unfortunately, resistance to these agents invariably develops after a median of 9–16 months^4^,^5^,^6^,^7^, and in ∼60% of patients resistance is mediated by selection for clones harbouring a secondary mutation in *EGFR* at position 790 (T790M)^8^,^9^,^10^,^11^. The third-generation covalent and mutant-selective EGFR TKIs rociletinib (CO-1686)^12^ and osimertinib (AZD9291)^13^ target both activating and T790M mutations, and have demonstrated activity in T790M-positive NSCLC patients^14^,^15^.

Although third-generation agents provide clinical benefit to many patients, some patients do not respond and complete responses are rare, suggesting that additional resistance mechanisms may decrease the efficacy of these inhibitors. Additionally, the mechanisms of resistance to these newer agents are not fully understood^16^,^17^,^18^. Initial findings in small patient cohorts have suggested that the dominant mechanisms of resistance to rociletinib and osimertinib may differ. However, both agents appear to lead to a preferential decrease of T790M-mutant cells^16^,^17^. While acquired resistance due to emergence of *EGFR* C797S mutations was observed in a significant fraction of osimertinib-treated patients^16^, acquired resistance to rociletinib was associated with *EGFR* amplification or histological transformation in a subset of patients^17^.

Overcoming tumour heterogeneity is a major challenge for the personalized treatment of cancer. Although intratumoural heterogeneity has been well described in a variety of cancer types^19^,^20^, including NSCLC^21^,^22^, the degree to which tumour heterogeneity currently influences treatment decisions in the clinic remains limited. Despite some evidence that multiple resistant subclones can arise following treatment of NSCLC patients with EGFR-targeted therapies^10^,^11^,^23^,^24^, the fraction of patients that develop multiple resistance mechanisms has not been systematically evaluated. This is due largely to the fact that prior studies have relied on tissue biopsies that are limited by the presence of geographic heterogeneity. Analysis of ctDNA has advantages over traditional biopsies in that the procedure is minimally invasive, is able to detect contributions from multiple tumour deposits, and can easily be repeated over time, allowing a more comprehensive analysis of tumour heterogeneity^25^,^26^,^27^.

Here, we employed ctDNA analysis using CAPP-Seq^28^,^29^ to study resistance to EGFR TKIs in T790M-mutant NSCLC patients treated with rociletinib. Since CAPP-Seq simultaneously assesses single-nucleotide variants (SNVs), insertions/deletions, rearrangements, and somatic copy-number alterations (SCNAs), it facilitates the broad exploration of potential resistance mechanisms. We found evidence for a high frequency of inter- and intra-patient heterogeneity of resistance mechanisms after initial EGFR TKI therapy and following rociletinib treatment. *EGFR* C797S, which arises in approximately one third of patients treated with the third-generation EGFR TKI osimertinib^16^, was observed in only one patient, suggesting that the pattern of resistance mechanisms to rociletinib and osimertinib differ. Increased *MET* copy number was the most frequently observed mechanism of rociletinib resistance and patients with multiple resistance mechanisms following initial EGFR TKI therapy (that is, both T790M and increased *MET* copy number) experienced inferior responses and significantly shorter progression-free survival (PFS) when treated with rociletinib. In agreement with these clinical findings, erlotinib-resistant xenografts treated with rociletinib reproducibly developed *MET* amplification. Importantly, sensitivity to rociletinib could be reinstated by combined therapy with the MET inhibitor crizotinib. Taken together, these results emphasize the clinical importance of intra-patient tumour heterogeneity arising during EGFR-targeted therapy for NSCLC.

## Results

### Overview of patient cohort

**Figure 1:**
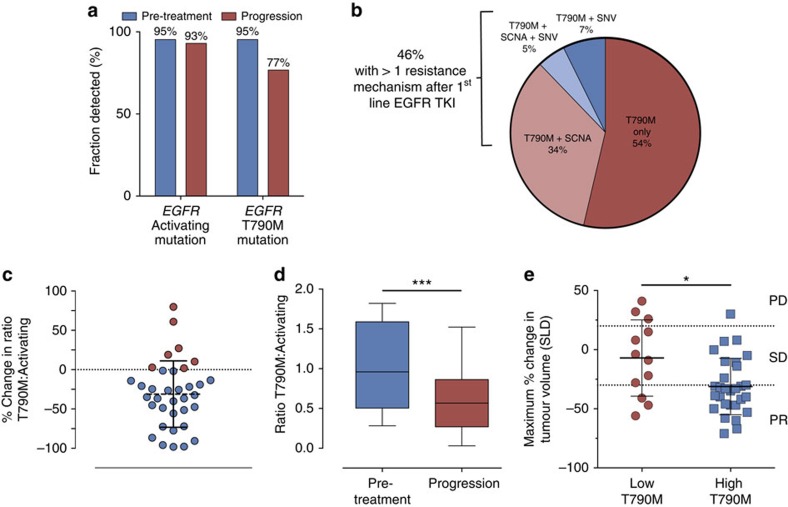
Heterogeneity of resistance mechanisms and *EGFR* mutation dynamics in response to EGFR TKIs. (**a**) Detection of *EGFR*-activating and T790M resistance mutations in pre-treatment and progression plasma samples from rociletinib-treated patients (*n*=43) using CAPP-Seq. (**b**) Summary of putative resistance mechanisms to first- and second-generation EGFR TKIs present in the pre-rociletinib plasma sample of patients with T790M mutations (*n*=41). (**c**) The percent change in the relative ratio of T790M to activating mutation alleles in progression plasma samples compared with pre-treatment samples from rociletinib-treated patients. Patients in whom the ratio decreased (*n*=28) are coloured blue and patients in whom the ratio increased (*n*=7) are coloured red. Only patients in whom both activating and T790M mutations were detectable pre-treatment, and activating mutations were detectable at progression are included. (**d**) Box and Whisker plots depicting the relative ratio of T790M and activating mutation alleles in pre-treatment and progression plasma samples from rociletinib-treated patients. The solid box represents the interquartile range of values and whiskers represent the 10th and 90th percentile values. All patients depicted in **c** are included. (*n*=35, ***P<0.0005, Wilcoxon signed-rank test). (**e**) Comparison of the ratio of T790M to *EGFR*-activating mutation in pre-treatment plasma samples and the best RECIST response to treatment with rociletinib. Patients with low baseline T790M (*n*=12, ratio ≤0.5) have significantly less reduction in tumour volume than patients with high baseline T790M (*n*=29, ratio > 0.5; * *P*<0.05, Wilcoxon rank-sum test). Only patients in whom both activating and T790M mutations were detectable pre-treatment are included.

To characterize potential mechanisms of resistance to first- and second-generation EGFR TKIs and rociletinib, we performed CAPP-Seq ctDNA profiling on 115 serial plasma samples from 43 patients included in phase 1 and 2 trials of rociletinib (Supplementary Table 1). All patients harboured activating mutations in *EGFR*, had progressed on at least one previous EGFR TKI therapy (77% were taking an EGFR inhibitor at the time of consent) and were T790M-Positive based on tissue and/or plasma testing. Demographic characteristics reflect a Western population with advanced NSCLC. Baseline blood samples were from screening or day 1 of cycle 1, and progression samples were chosen based on the date of systemic progression (Methods). We employed a 302 kb CAPP-Seq selector targeting 252 genes recurrently mutated in NSCLC (Supplementary Table 2). *EGFR*-activating mutations were detected in 95% and 93% of pre-treatment and progression plasma samples, respectively, while T790M was detected in 95% and 77% of pre-treatment and progression plasma samples (Fig. 1a). The concordance between tissue and plasma genotyping for *EGFR* activating and T790M mutations in pre-treatment tumour biopsies and plasma was 95% (41 of 43) and 91% (39 of 43), respectively.

### Heterogeneity of resistance mechanisms to initial EGFR TKIs

Prior studies analysing tissue biopsies from patients following progression on first- and second-generation EGFR TKIs have reported the presence of multiple resistance mechanisms in ∼5–15% of cases^10^,^11^,^23^,^24^. However, two limitations of these studies are the analysis of a single tumour deposit, and that sufficient tissue to perform all of the assays was not always available. Since ctDNA profiling integrates contributions from many tumour deposits, it has the potential to more accurately identify geographic heterogeneity of resistance mechanisms. Indeed, among the 41 patients who had detectable T790M mutations at enrolment following prior EGFR-targeted therapies, additional putative resistance mutations were detected in the pre-treatment plasma from 19 (46%; Fig. 1b). In addition to T790M, 14 patients (34%) harboured an increased copy number in *MET* or *ERBB2*, three patients (7%) harboured one or more additional SNVs in *EGFR*, *PIK3CA* or *RB1*, and two patients (5%) harboured both an increased copy number in *MET* and a SNV in *PIK3CA* or *RB1.* Thus the co-occurrence of T790M with other resistance mechanisms to first-line EGFR TKIs is considerably greater than previously reported and such intra-patient heterogeneity may impact the clinical response to subsequent EGFR TKIs such as rociletinib.

### Rociletinib selectively targets T790M-containing subclones

In 28 out of 35 patients in whom T790M was detectable pre-treatment and activating mutations were present at both time points, the relative ratio of T790M to activating mutation decreased at progression (Fig. 1c). Similarly, considering all patients, the ratio of T790M to activating mutation was significantly lower at progression compared to pre-treatment (Fig. 1d, *P*<0.0005, Wilcoxon signed-rank test). Based on the preferential elimination of T790M-containing subclones by rociletinib, we hypothesized that the ratio of T790M to *EGFR*-activating mutation prior to treatment may predict response to rociletinib. Indeed, this ratio was modestly but significantly correlated with best response to therapy (Spearman rho=0.35, *P*<0.05) and patients in whom the ratio of T790M to activating mutation was ≤0.5 (*n*=12, low T790M) experienced significantly less tumour shrinkage than the remaining patients (*n*=29, high T790M; Fig. 1e, average change in target lesions using Response Evaluation Criteria In Solid Tumors (RECIST) methodology^30^ 7.1 versus −31.2%; *P*<0.05, Wilcoxon rank-sum test). A relative decrease of T790M was similarly observed in a subset of progression biopsy tissue specimens collected from rociletinib-treated patients^17^. Thus, rociletinib selectively targets T790M-containing subclones and ctDNA recapitulates changes in tumour heterogeneity observed in tissue biopsies.

### Heterogeneity of resistance mechanisms to rociletinib

**Figure 2:**
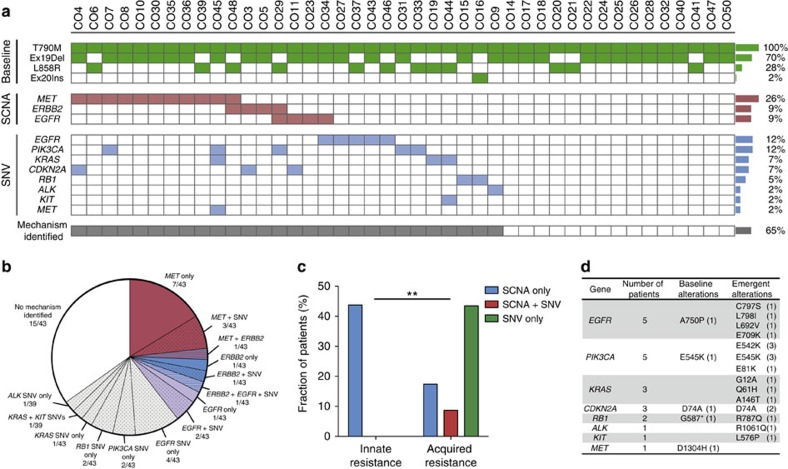
Inter- and intra-patient heterogeneity of resistance mechanisms to rociletinib. (**a**) Summary of putative resistance mechanisms to rociletinib identified by CAPP-Seq. T790M and *EGFR*-activating mutations detected in plasma at one or more time points are indicated in green (*n*=43). Only alterations that were emergent or increased in relative abundance following therapy are shown. Somatic copy-number alteration (SCNA) and single-nucleotide variant (SNV) identification was not feasible in all plasma samples due to low or undetectable ctDNA levels. (**b**) Summary of putative resistance mechanisms to rociletinib organized by gene. Red, blue and purple signify the presence of a SCNA affecting *MET*, *ERBB2* or *EGFR*, respectively. Grey signifies the presence of one or more SNVs in patients without SCNAs. Striped shading represents the concurrent presence of multiple SCNA's, black dots represent the presence of one or more SNVs, and solid white indicates patients in whom no mechanism was identified. (**c**) Comparison of putative resistance mechanisms in patients with innate and acquired resistance to rociletinib. Patients with innate resistance (*n*=15) were defined as those with progression-free survival (PFS) of less than 3 months, and patients with acquired resistance were defined as those with PFS of greater than 3 months (*n*=28). The mechanisms identified were significantly different in patients with innate versus acquired resistance (**P<0.005; Fisher's exact test). (**d**) Baseline and emergent SNVs detected by CAPP-Seq in the plasma of patients treated with rociletinib. Only SNVs that were emergent or increased in relative abundance following therapy are shown. The number of patients with each specific variant is indicated in parentheses.

To identify mechanisms of resistance to rociletinib, we reasoned that aberrations in genes that drive resistance should be positively selected for over the course of therapy. Accordingly, we identified alterations that were absent before treatment but emerged at progression or that increased in relative abundance over the course of therapy (Methods). Using this approach, we identified one or more putative resistance mechanisms in 28 out of 43 patients (65%; Fig. 2a). *MET* copy-number gain was the most frequent mechanism and was observed in 11 patients (26%; Fig. 2b). Furthermore, eight patients (19%) displayed putative resistance mechanisms affecting multiple genes, including the concurrent presence of both SNVs and SCNAs in seven patients (16%). Thus, as with earlier generations of EGFR TKIs, a significant fraction of patients develop multiple resistance mechanisms to rociletinib, reflecting the clinical importance of tumour heterogeneity.

We next sought to explore differences between mechanisms of innate and acquired resistance. Patients with innate resistance were defined as those with PFS shorter than 3 months, while patients with a PFS longer than 3 months were considered as having acquired resistance (Methods). Interestingly, we observed distinct patterns of genomic alterations in patients with innate versus acquired resistance. Specifically, pre-existing copy-number gains in *MET, ERBB2* and *EGFR* were significantly more common in patients with innate resistance (*n*=15), and conversely, emergent or increasing SNVs were more common in patients with acquired resistance (*n*=28; Fig. 2c, *P*<0.005, Fisher's exact test). This suggests that detection of copy-number gains leading to EGFR bypass pathway activation before third-generation EGFR TKI therapy may allow identification of patients likely to harbour innate resistance.

Among the 43 patients, 18 were identified as having one or more SNVs as a putative rociletinib resistance mechanism. In this context, mutations in *PIK3CA* and *EGFR* were the second most recurrent mechanism of resistance overall, each occurring in five patients (12%; Fig. 2d, Supplementary Fig. 1). Two activating mutations in *PIK3CA* (E542K and E545K) were frequently observed, occurring in three and four different patients, respectively (Supplementary Fig. 2). Four patients also displayed emergent mutations in *EGFR* (E709K, L692V, C797S and L798I), although no recurrent mutations were observed. Intriguingly, we also observed the emergence of distinct *KRAS* activating mutations (G12A, Q61H and A146T) in three patients, none of whom had evidence of these lesions in their pre-treatment plasma specimens. While it is well established that *KRAS* activation is a mechanism of acquired resistance in colorectal cancer patients treated with EGFR-targeting monoclonal antibodies (mAbs)^25^,^26^,^31^,^32^, this is to our knowledge the first report of *EGFR* mutant NSCLC patients acquiring activating mutations in *KRAS* following treatment with an EGFR TKI^10^,^11^,^23^.

**Figure 3:**
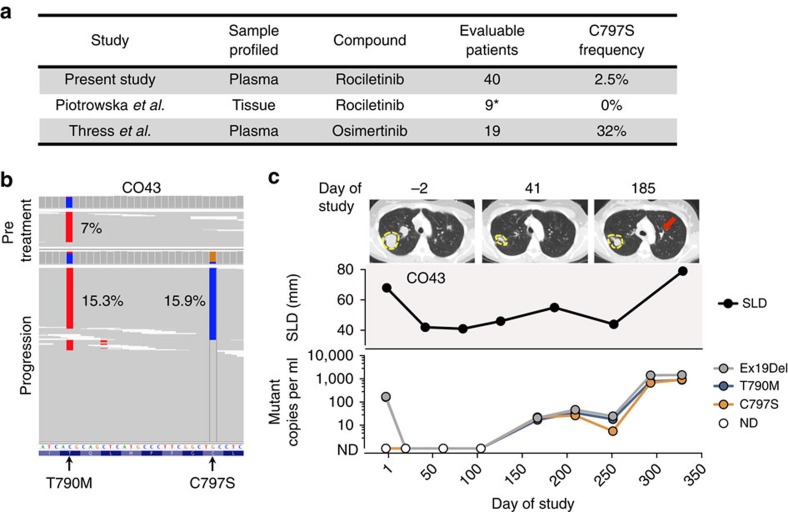
*EGFR* C797S is an infrequent mechanism of rociletinib resistance. (**a**) The prevalence of *EGFR* C797S mutations reported in post-treatment tissue biopsies or plasma samples following treatment with the third-generation EGFR TKIs rociletinib^17^ and osimertinib^16^. Only patients with detectable *EGFR*-activating mutations in progression tissue or plasma are considered (*=only patients unique to the Piotrowska *et al*. study were included). (**b**) An acquired *EGFR* C797S mutation was observed in *cis* with T790M in progression plasma from CO43. The allele fraction of each mutation in pre-treatment and progression plasma is shown. (**c**) Serial tumor and ctDNA measurements from CO43. Representative CT scans at the time points indicated are provided; the largest target lesion is outlined and the emergence of a new lesion is indicated with an arrow. The upper panel displays the tumor volume represented by the sum of longest diameters (SLD) of target lesions. The lower panel displays alterations in *EGFR* detected in plasma. ND, not detected.

The *EGFR* C797S mutation is known to prevent the covalent binding of all irreversible EGFR TKIs^16^,^33^,^34^, and has recently been observed as the most common mechanism of acquired resistance to osimertinib, occurring in 32% of patients^16^. In contrast, considering the present study and a prior study evaluating post-progression tissue biopsies from rociletinib-treated patients^17^, the C797S mutation has been observed in a total of one out of 49 (2%) evaluable patients who progressed on rociletinib (Fig. 3a). Thus, the pattern of resistance mechanisms to rociletinib and osimertinib appear to differ. In CO43 an emergent C797S mutation was observed in *cis* with the T790M mutation at progression (Fig. 3b) and was present at an allele fraction similar to that of T790M and the activating mutation (Fig. 3c), suggesting that this mutation is the dominant driver of resistance in this patient.

**Figure 4:**
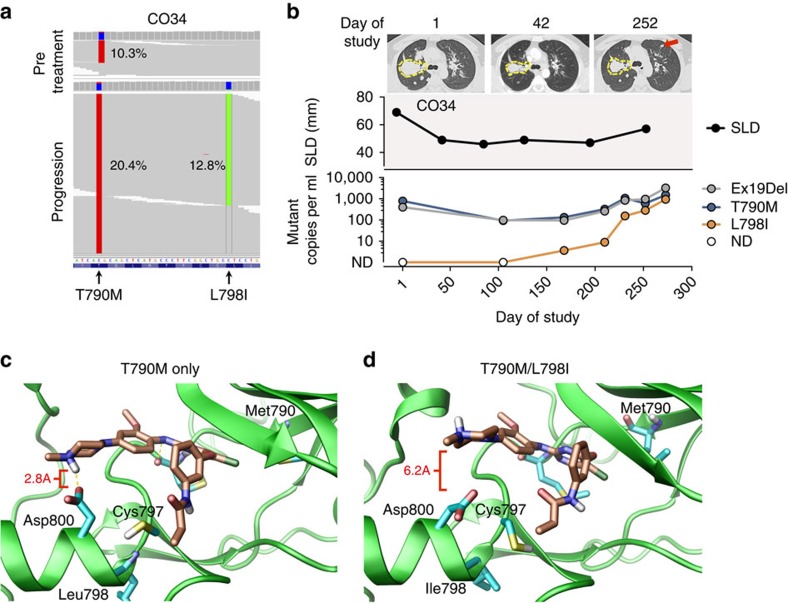
*EGFR* L798I mediates rociletinib resistance. (**a**) An acquired *EGFR* L798I mutation was observed in *cis* with T790M in progression plasma from CO34. The allele fraction of each mutation in pre-treatment and progression plasma is shown. (**b**) Serial tumour and ctDNA measurements from CO34. Representative CT scans at the time points indicated are provided; the largest target lesion is outlined, and the emergence of a new lesion is indicated with an arrow. The upper panel displays the tumour volume represented by the sum of longest diameters (SLD) of target lesions. The lower panel displays alterations in *EGFR* detected in plasma. ND, not detected. (**c**,**d**) Structural modelling of rociletinib binding to (**c**) EGFR^T790M^ and (**d**) EGFR^T790M/L798I^. The EGFR kinase is shown in a ribbon representation (green) with rociletinib in orange. Hydrogen bonding (yellow dashed lines) between the Asp800 residue in EGFR^T790M^ and the quaternary piperazine NH+ of rociletinib is disrupted in the EGFR^T790M/L798I^ mutant as a result of increased separation (average distance of 2.8A versus 6.2A in EGFR^T790M^ and EGFR^T790M/L798I^, respectively; red brackets). In the EGFR^T790M/L798I^ mutant the orientation of Cys797 for reactivity with rociletinib is less optimal due to the loss of stabilizing interactions and other subtle angle changes.

We also identified a novel *EGFR* L798I mutation in one patient (CO34). Similar to the pattern of emergence of C797S in CO43, the L798I mutation was found to occur in *cis* with T790M (Fig. 4a), and the relative allele fraction of the mutation compared with the dominant clone increased over the course of therapy (Fig. 4b), consistent with this alteration driving resistance. The L798 residue is directly adjacent to the covalent binding site of rociletinib (C797) and this mutation has not been previously observed *in vitro* or in patients treated with EGFR TKIs^10^,^11^,^16^,^17^,^23^,^33^,^34^,^35^,^36^,^37^. Given the potential of the L798I mutation to alter the interaction between rociletinib and its binding site, we performed *in silico* modelling of this mutation (Methods). Molecular dynamics simulations indicated that the L798I mutation affects the local orientation of nearby side chains critical for rociletinib positioning and covalent bond formation at C797. Specifically, in EGFR^T790M^, the Asp800 sidechain is positioned to H-bond to the quaternary piperazine NH+ of the ligand (Fig. 4c), whereas the ligand orientation in EGFR^T790M/L798I^ is rotated in this region and this H-bond likely does not form (Fig. 4d). This likely reduces the affinity of ligand binding necessary for reactivity. Additionally, the angle of the Cys797 sidechain is altered, likely diminishing its nucleophilicity.

### *MET* mediates innate and acquired resistance to rociletinib

**Figure 5:**
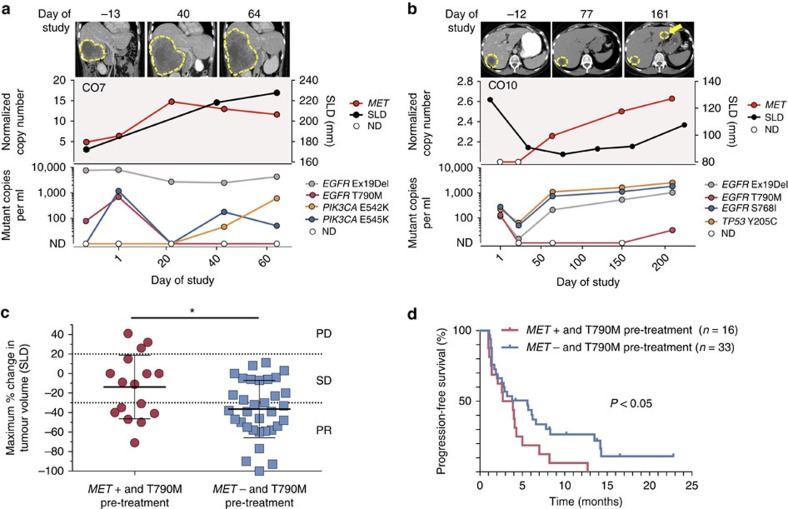
*MET* copy-number gain mediates innate and acquired resistance to rociletinib. (**a**,**b**) Representative vignettes of patients with innate (**a**) and acquired (**b**) resistance to rociletinib. The upper panel displays representative CT scans at the time points indicated; lesions are outlined, and the emergence of a new lesion is indicated with an arrow. The middle panel displays the copy number of *MET* detected in plasma normalized by % ctDNA and the tumour volume represented by the sum of longest diameters (SLD) of target lesions. The lower panel displays SNVs detected in plasma. ND, not detected. (**c**) Scatter plot showing best RECIST response following rociletinib treatment in patients whose tumours had both T790M mutations and *MET* copy-number gain detected in tissue or plasma prior to rociletinib treatment (red; *n*=16) and those with T790M-mutant tumours confirmed negative for *MET* amplification by FISH (blue; *n*=33; * *P*<0.05, Wilcoxon rank-sum test). The mean and standard deviation are indicated with a solid line and whiskers, respectively. (**d**) Kaplan–Meier plot of progression-free survival of patients with T790M-mutant tumours with (red; *n*=16) or without (blue; *n*=33) evidence of *MET* copy-number gain prior to rociletinib treatment. Progression was defined according to RECIST 1.1 methodology. Patients alive without progression by RECIST 1.1 were censored at their last evaluable radiologic disease assessment date before the data-cutoff date. Small vertical lines represent censored events; statistical significance was assessed using a log-rank (Mantel–Cox) test (*P*<0.05).

Dynamic changes in competing subclones were apparent during rociletinib therapy in many patients. For example, in CO7 a subclone with *MET* copy-number gain detected before treatment increased over the course of therapy while the abundance of two different activating *PIK3CA* mutations varied over time (Fig. 5a). In CO10, a subclone with *MET* copy-number gain emerged during rociletinib therapy and then increased continuously while T790M initially disappeared and later re-emerged (Fig. 5b). Interestingly, a new liver metastasis was detected at progression and we hypothesize that this deposit may harbour the subclone with *MET* copy-number gain. In both CO7 and CO10, the normalized copy number of *MET* in ctDNA paralleled increasing tumour burden over the course of treatment, suggesting that *MET* copy-number gain is mediating resistance in these patients.

To further investigate *MET* as a mechanism of resistance to rociletinib, we identified an expanded cohort of 16 patients with T790M-mutant tumours participating in rociletinib trials who had *MET* copy-number gain detected in pre-treatment biopsies or plasma (Methods). Patients whose tumours had both T790M mutations and *MET* copy-number gain before rociletinib treatment (*n*=16) displayed significantly less tumour shrinkage than patients whose tumours were T790M-mutant but lacked *MET* amplification by FISH (*n*=33; Fig. 5c, average change in target lesions using RECIST methodology of −13.8 versus −36.5%; *P*<0.05, Wilcoxon rank-sum test). Patients with pre-treatment *MET* gains also had a significantly shorter median PFS than patients without *MET* alterations (Fig. 5d, 3.3 months versus 5.6 months; *P*<0.05, log-rank test). These findings indicate that the presence of multiple resistance mechanisms following initial EGFR TKI therapy (that is, concomitant T790M mutations and *MET* copy-number gain) is associated with an inferior therapeutic response to rociletinib.

We also observed recurrent copy-number gains in *EGFR* and *ERBB2*, two key ErbB-family members. Accordingly, we sought to determine whether overexpressing these genes would induce rociletinib resistance in the context of *EGFR* mutations. NCI-H1975 cells, which harbour the *EGFR* L858R activating mutation, as well as the T790M resistance mutation, were transfected with lentiviral expression vectors encoding EGFR or ERBB2 and sensitivity to rociletinib was assessed using growth inhibition assays. Both EGFR and ERBB2 overexpression significantly reduced the potency of rociletinib, by 2.2- (*P*<0.05, Wilcoxon rank-sum test) and 3.7-fold (*P*<0.0005, Wilcoxon rank-sum test), respectively (Supplementary Fig. 3).

### Preclinical models of rociletinib resistance

**Figure 6:**
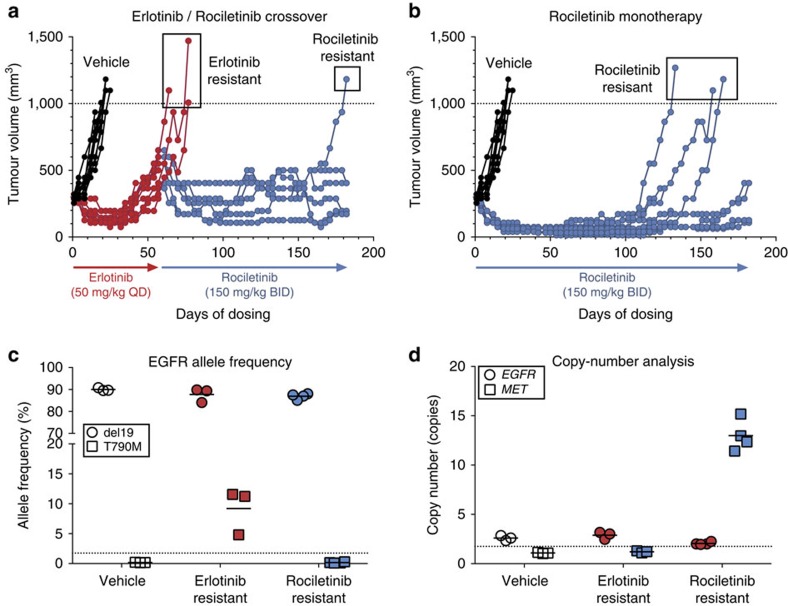
*MET* amplification arises in xenograft models of acquired resistance to rociletinib. (**a**,**b**) Mice bearing PC-9 NSCLC xenograft tumours (Ex19Del) were treated with the compounds, doses and schedules indicated (*n*=10 per group). Each line represents an individual animal. On day 60 the erlotinib-treated cohort was split into 2 groups and 3 animals continued on erlotinib while the rest (*n*=7) were crossed-over to rociletinib. The mean tumour volume in erlotinib monotherapy, erlotinib crossover, and rociletinib monotherapy treated animals was compared using the Wilcoxon rank-sum test. (**c**,**d**) Next-generation sequencing was used to assess (**c**) Ex19Del and T790M allele frequencies and (**d**) *EGFR/MET* copy number in the tumours collected at endpoint from mice in the vehicle (*n*=3), erlotinib resistant (*n*=3) and rociletinib-resistant (*n*=4) groups.

**Figure 7:**
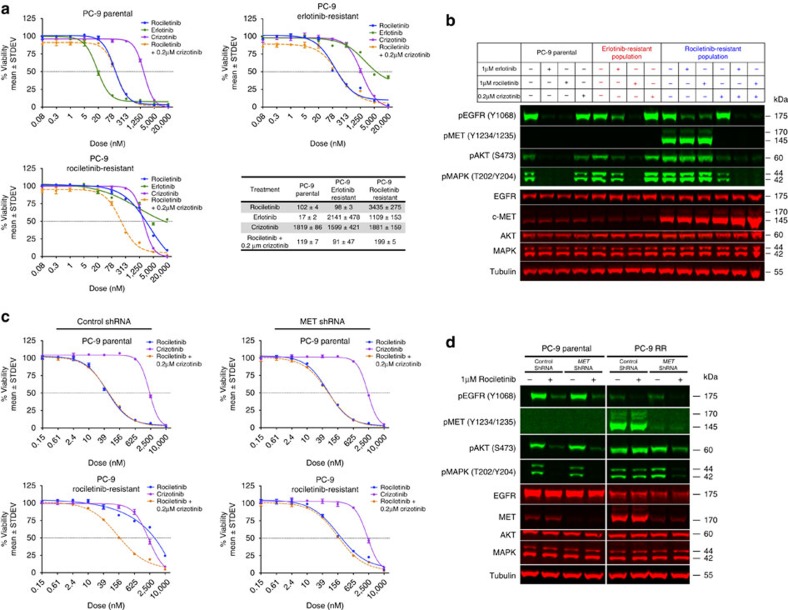
The MET inhibitor crizotinib restores rociletinib sensitivity and downstream pathway suppression. (**a**) Erlotinib resistant (ER) and rociletinib-resistant (RR) tumour-derived cell lines respond to rociletinib and rociletinib combined with crizotinib, respectively. Cell viability was evaluated in ER and RR tumour-derived cell lines after treatment with erlotinib, rociletinib and/or crizotinib for 72 h. Experiments were performed in triplicate, repeated three times and data are plotted as mean percentage viability±s.d. relative to control. The data summary table reflects these 3 experiments and the values reported are the mean 50% growth inhibition±s.d. (nM). (**b**) Western blot analysis of PC-9 parental, erlotinib resistant, and rociletinib-resistant cell lines following 1-h incubations with the compounds indicated. (**c**) PC-9 parental and rociletinib-resistant tumour-derived cell lines were infected with a lentivirus expressing MET-specific or scrambled control shRNAs. Cell viability was evaluated after treatment with rociletinib and/or crizotinib for 72 h. Data are plotted as mean percentage viability±s.d. relative to control. (**d**) Western blot analysis of PC-9 parental and rociletinib-resistant (RR) cell lines transfected with control or MET shRNA and treated with rociletinib.

In parallel with our clinical observations, we also characterized mechanisms of resistance to rociletinib using *in vivo* preclinical models. PC-9 cells harbour the common *EGFR*-activating exon 19 deletion delE746-A750 and are sensitive to first-, second- and third-generation EGFR inhibitors^12^. PC-9 tumour-bearing mice were treated with clinically relevant doses of erlotinib or rociletinib. Although both treatments resulted in an initial reduction in tumour growth, tumours in the erlotinib-treated cohort increased in size after day 32. By day 57, the mean tumour volume in erlotinib-treated animals was ninefold larger than in rociletinib-treated littermates (Fig. 6a,b, *P*<0.001, Wilcoxon rank-sum test). Erlotinib-resistant (ER) tumours arising in these mice reproducibly acquired *EGFR* T790M *in vivo*, while rociletinib monotherapy prevented the emergence of T790M clones and maintained tumour stasis for ∼100 days (Fig. 6c). Growth inhibition assays and immunoblot profiling confirmed erlotinib resistance *in vitro* (Fig. 7a,b). Consistent with findings in rociletinib-treated patients, these data demonstrate that the relative ratio of T790M to activating mutation decreases as a direct result of negative clonal selection by rociletinib.

To mimic the treatment of patients with rociletinib following progression on a first-generation EGFR TKI, mice progressing on erlotinib were separated into two cohorts on day 60. Three mice continued erlotinib treatment while the seven remaining mice were crossed-over to rociletinib. In the crossover cohort, rociletinib treatment resulted in a significant reduction in tumour volume (Fig. 6a, *P*<0.01, Wilcoxon rank-sum test). Rociletinib dosing was continued in the monotherapy and crossover groups until resistance emerged, at which time individual tumours were collected and analysed. CAPP-Seq profiling of rociletinib-resistant (RR) tumours identified *MET* amplification as the sole somatic aberration emergent in RR tumours when compared to vehicle treated tumours (Fig. 6d). Notably, all four RR tumours were T790M-negative. Next-generation sequencing, growth inhibition assays, receptor tyrosine kinase arrays, FISH and immunoblot profiling confirmed the acquisition of *MET* amplification (11–15 copy gain) and MET pathway activation as the mechanism of acquired resistance (Fig. 6c,d; Fig. 7a,b; Supplementary Fig. 4).

**Figure 8:**
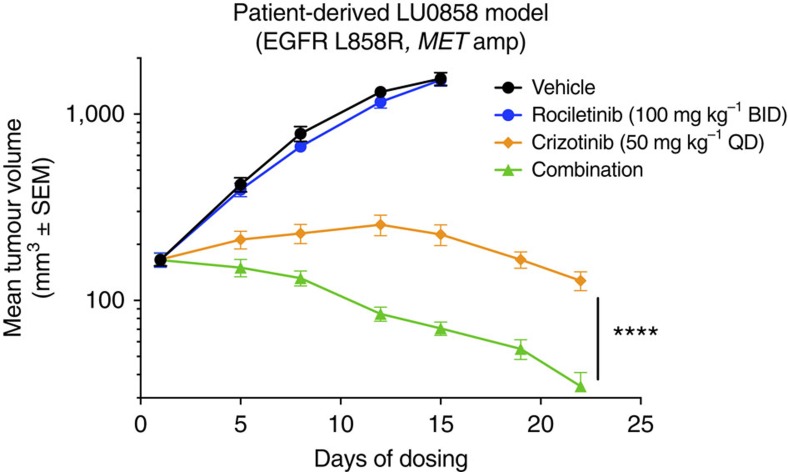
The MET inhibitor crizotinib restores rociletinib sensitivity in a patient-derived NSCLC xenograft model of innate rociletinib resistance. Mice bearing LU0858 NSCLC patient-derived xenograft tumours (L858R mutant and 14-copy *MET* amplification) were orally administered rociletinib, crizotinib or the combination using the doses and schedules indicated (*n*=10 per group). The endpoint tumour volumes between the crizotinib and combination groups on day 22 were compared using a paired two tailed student's *t*-test (*n*=10; ****P<0.0001).

Given these observations, we hypothesized that combination therapy targeting both EGFR and MET could overcome bypass pathway activation and drug resistance. When we concurrently treated RR cells with rociletinib and the MET inhibitor crizotinib, rociletinib sensitivity and downstream pathway suppression were restored (Fig. 7a,b). Similarly, MET knockdown using a lentivirus expressing MET-specific shRNA also restored rociletinib sensitivity and downstream pathway suppression in RR cells (Fig. 7c,d). Notably, the combination of rociletinib and crizotinib was also able to restore activity of rociletinib in a patient-derived NSCLC xenograft model that harbours an *EGFR* L858R activating mutation and 14-copy amplification of *MET* (Fig. 8; Supplementary Fig. 5). These data support a model in which resistance to erlotinib and rociletinib in PC-9 cells results from expansion of subpopulations of EGFR del19/T790M and del19/MET-amplified subclones, respectively.

## Discussion

Collectively, in performing ctDNA analysis using CAPP-Seq we observed a previously unrecognized high frequency of molecular heterogeneity in resistance mechanisms following treatment of NSCLC patients with front-line and with third-generation EGFR TKIs. Importantly, we found that patients who developed multiple resistance mechanisms following initial EGFR TKI therapy (T790M and increased *MET* copy number) displayed inferior responses and significantly shorter progression-free survival when treated with rociletinib. While prior studies have observed intra-patient heterogeneity of resistance mechanisms in ∼5–15% of *EGFR* mutant NSCLC patients^10^,^11^,^23^,^24^, we found evidence for multiple resistance mechanisms in 46% of T790M-mutant patients. These findings underscore the clinical relevance of tumour heterogeneity in these patients since it is likely that targeting T790M alone will sub-optimally treat patients whose tumours display multiple resistance mechanisms. This heterogeneity also highlights a significant advantage of ctDNA analysis over tissue biopsies, given its ability to simultaneously capture and noninvasively detect mutations present in multiple tumour deposits.

We observed a number of previously unreported resistance mechanisms to EGFR TKIs in NSCLC patients, including a novel tertiary mutation in *EGFR* (L798I) and the emergence of activating *KRAS* mutations in patients following rociletinib therapy. Mutations in *EGFR* and *KRAS* are considered to be mutually exclusive in NSCLC^38^, although rare exceptions at diagnosis have been described^39^,^40^,^41^. *De novo KRAS* mutations are associated with decreased responsiveness to EGFR-targeting TKIs in NSCLC and mAbs in colorectal cancer patients^42^,^43^. While *KRAS* activation is a known mechanism of acquired resistance to mAb-based EGFR blockade in colorectal cancer patients^25^,^26^,^31^,^32^, to our knowledge, this is the first report of emergent *KRAS* alterations in NSCLC following treatment with an EGFR-directed therapy^10^,^11^,^23^.

Significantly, our findings suggest that the pattern of resistance mechanisms to third-generation EGFR TKIs appear to be drug-specific, differentiating these compounds from first-generation EGFR TKIs for which T790M mutations predominate regardless of which inhibitor is used. While *EGFR* C797S mutations arise in approximately one third of patients treated with osimertinib^16^, this mutation has only been observed in ∼2% of patients (one out of 49) treated with rociletinib. The difference in the frequency of C797S mutations in patients treated with rociletinib and osimertinib may be due to differing potencies or pharmacokinetics of the two drugs, as well as potential off-target activities.

Understanding the mechanisms of response and resistance to these agents may inform strategies for combining or sequencing them to provide patients with the greatest clinical benefit. For example, although patients treated with a prior T790M-targeted agent were excluded from our study, a recent report in a small number of patients suggests that sequencing osimertinib after rociletinib may provide additional clinical benefit to patients^44^. This could potentially allow patients with T790M-mutant tumours to remain on an EGFR TKI longer by delaying the emergence of C797S, which prevents the covalent binding of all irreversible EGFR TKIs^16^,^33^,^34^. This scenario may be analogous to observations in NSCLC patients harbouring *ALK* rearrangements, in whom the next-generation ALK inhibitor ceritinib can induce responses in patients who developed resistance to the less potent inhibitor crizotinib^45^,^46^. Thus, rational sequencing of drugs with different patterns of resistance mechanisms may be a generalizable strategy for maximizing therapeutic benefits.

Contrary to our findings in patients, preclinical studies have suggested that resistance to third-generation mutant-selective EGFR TKIs such as rociletinib would primarily involve additional mutations in *EGFR* itself (for example, C797S)^33^,^34^, rather than bypass pathway activation. Instead, we found *MET* copy-number gain to be the most highly recurrent resistance mechanism to rociletinib (observed in 26% of patients). This observation points to the importance of comprehensive resistance mechanism profiling in patient specimens.

The prevalence of *MET* copy-number gain observed in this cohort is at the upper end of previously reported values, which range from 5 to 20% in NSCLC patients progressing on first-line EGFR TKIs^10^,^11^,^47^,^48^. Likely contributions to the range of *MET* prevalence reported in prior studies include variability in the cytogenetic methods used, and the fact that not all patients were tested for all aberrations due to limited tissue. Additionally, the majority of prior studies have focused on tissue biopsies which likely under-estimate spatial and temporal heterogeneity in resistance mutations within individual patients^21^,^22^. Previous studies that have examined ctDNA in NSCLC patients with resistance to EGFR TKIs have primarily used techniques limited to interrogating mutations in *EGFR*^16^,^49^,^50^,^51^,^52^,^53^,^54^, and therefore were unable to detect SCNAs in bypass pathway genes such as *MET*.

The CAPP-Seq selector used in this study was not designed to distinguish between focal gene amplifications of *MET* and polysomy of chromosome 7. However, we observed many examples of SCNAs involving either *MET* or *EGFR* but not both genes (78% of plasma samples with SCNAs in *MET* or *EGFR* demonstrated this pattern), suggesting focal gene amplifications in these syntenic genes rather than polysomy. Separately, in those patients in whom SCNAs in both *MET* and *EGFR* were detectable in ctDNA before rociletinib, therapeutic resistance to rociletinib was almost invariably associated with selection for *MET* alone (by exhibiting further gains in *MET* copy number, but not *EGFR*). These observations, along with our findings in preclinical models, strongly suggest that focal SCNAs involving *MET*, but not chromosome 7 polysomy, represent the prevailing resistance mechanism to rociletinib. Nevertheless, chromosome 7 polysomy harbours prognostic value in the context of EGFR TKI therapy in NSCLC patients^55^,^56^,^57^,^58^ and therefore has potential therapeutic relevance in this patient population.

In summary, our results demonstrate that noninvasive profiling of resistance mechanisms using ctDNA analysis can define patterns of resistance to targeted therapy. These findings have important implications for selection of patients most likely to respond to single pathway inhibition as well as for the design of clinical studies attempting to overcome intrinsic and acquired resistance. For example, our findings that concurrent treatment with crizotinib can re-sensitize *EGFR*-mutant tumours with *MET* amplification to rociletinib suggest that personalized targeting of multiple resistance mechanisms may be of significant clinical utility. We envision that in the near future ctDNA analysis will be used to detect both pre-existing and emergent resistance mutations to identify rational combination therapies that target tumour heterogeneity-driven resistance in a personalized fashion.

## Methods

### Trial design and patient selection for ctDNA profiling

All of the patients evaluated in this manuscript were enrolled in NCT01526928 (Study to Evaluate Safety, Pharmacokinetics, and Efficacy of Rociletinib (CO-1686) in previously treated mutant EGFR NSCLC patients) or NCT02147990 (TIGER-2: A phase 2, open-label, multicenter, safety and efficacy study of oral CO-1686 as 2nd line EGFR-directed TKI in patients with mutant EGFR NSCLC). Patients received oral rociletinib in 21-day continuous cycles until disease progression according to the RECIST version 1.1, unacceptable toxic effects, or withdrawal of consent occurred. Treatment beyond progression was permitted if the investigator believed the patient was still benefiting. The study protocols were approved by the Institutional Review Board at participating centres, and all patients provided informed consent before treatment.

Patients were initially triaged for ctDNA profiling based on dose level. The majority (41/43) of patients were treated at therapeutic dose levels, defined as 900 mg rociletinib FB or all HBr dose levels, however two additional patients at lower dose levels were also included based on their clinical profiles^15^. The majority (41/43) of patients were T790M positive by tissue testing, however all patients were T790M positive by tissue and/or plasma ctDNA analysis. Patients with systemic progression were selected for ctDNA profiling based on availability of pre-treatment and post-progression plasma samples. Clinical analyses were performed using interim data from ongoing rociletinib trials and the data-cutoff date for the patients reported in this manuscript was 18 September 2015.

Demographic characteristics were typical of patients with advanced *EGFR*-mutated NSCLC (Supplementary Table 1). Of the 43 patients evaluated here, 16 were male and 27 were female, they ranged in age from 30 to 84 (median of 56), and 75% of the patients were white. The median number of prior treatments was 4, all patients had received at least one previous line of EGFR tyrosine kinase inhibitor therapy (most frequently erlotinib), and 77% of the patients were taking an EGFR inhibitor at the time of consent.

### Expanded *MET*-amplified cohort

To allow us to assess the relationship between *MET* copy-number gain and response to treatment with rociletinib (Fig. 5c,d), we identified a total of 16 patients with T790M-mutant tumours who had *MET* copy-number gain identified before rociletinib treatment by: (1) CAPP-Seq ctDNA plasma profiling (11 patients); (2) a positive *MET* FISH result on the pre-rociletinib treatment biopsy sample (3 patients); (3) documented *MET* amplification in patient history before trial enrolment (2 patients). The average change in target lesions using RECIST methodology and progression-free survival of patients with evidence of *MET* copy-number gain and/or amplification before treatment with rociletinib was then compared with a cohort of patients determined to be negative for *MET* amplification by FISH (‘*MET* negative'). Progression was defined according to RECIST 1.1 methodology^30^. Patients alive without progression by RECIST 1.1 were censored at their last evaluable radiologic disease assessment date before the data-cutoff date. The *MET*-negative patients were identified from a larger cohort of 33 patients with T790M-mutant tumours dosed with rociletinib at therapeutic dose levels. FISH testing was performed by Colorado Molecular Correlates Laboratory (Aurora, CO) and *MET* amplification positive cases were required to have one or more of the following features: (1) gene-to-centromere (*MET*/CEP7) ratio≥ 2.0; (2) mean number of *MET* signals per tumour cell nucleus ≥6; (3) percentage of tumour cells containing ≥15 *MET* signals or large clusters ≥10%.

### Cell-free DNA analysis by CAPP-Seq

Blood was collected at baseline, treatment day 15, and at the start of each 21-day cycle. Up to 24 ml of blood was collected in K_2_EDTA tubes, processed into plasma within 15±5 min (1,800*g* for 10 min at 18–23 °C), pooled and stored at −80 °C in 2.2 ml aliquots until cfDNA isolation. Circulating tumour DNA analysis was performed using Cancer Personalized Profiling by deep sequencing (CAPP-Seq) as previously described^28^,^29^. Briefly, cell-free DNA (cfDNA) was extracted from 2 to 4 ml (average of 3.44 ml) of plasma using the QiaAmp Circulating Nucleic Acid Kit (Qiagen, cat. no. 55114) according to manufacturer's instructions. Following isolation, DNA was quantified using the Qubit dsDNA High Sensitivity Kit (Life Technologies, cat. no. Q32851). A maximum of 32 ng of cfDNA was input into sequencing library preparation (if less than 32 ng was available all the cfDNA obtained was input into library preparation). Sequencing library preparation was performed using the KAPA LTP Library Prep Kit (Kapa Biosystems, cat. no. KK8232) with some modifications to the manufacturer's protocol.

Following sequencing library preparation, hybridization-based enrichment of specific sequences was performed using a custom designed pool of biotinylated DNA oligonucleotides (Roche NimbleGen). The regions targeted for sequencing were determined as previously described^28^. Briefly, an iterative algorithm was applied to Cosmic, TCGA, and other data sources to identify recurrently mutated regions of the genome in NSCLC. The regions targeted for sequencing are referred to as the ‘selector', and the algorithm utilized by CAPP-Seq is optimized to maximize the number of mutations targeted per patient while minimizing sequencing space (e.g. selector size).

Two NSCLC selectors were utilized by this study. Plasma samples were first profiled using a ∼170 kb selector in order to assess ctDNA burden. Patients with detectable ctDNA were then re-captured using a ∼302 kb selector for more comprehensive mutation profiling. The NSCLC selector targets 771 non-contiguous regions of the human genome, covering 302 kb across 252 genes (Supplementary Table 2). Regions targeted include recurrently mutated exons of potential driver genes (for example, *KRAS, MYC, PTEN, TP53* and *RB1*) as well as the entire kinase domain of the receptor tyrosine kinases *ALK, BRAF, EGFR, ERBB2, MET, PIK3CA, RET* and *ROS1*.

Following hybridization-based enrichment samples were sequenced on an Illumina HiSeq 2500 High Output lane to a median depth of ∼5,500 ×. Sequencing data were processed using a custom bioinformatics pipeline and SNV, indel and somatic copy-number alteration calling was performed as previously described^28^,^29^. SCNA analysis was performed using an approach described in detail below. Patient samples analysed in our study were collected under an IRB that did not allow for using germline DNA for genetic analyses. To distinguish between germline variants and somatic alterations, we applied a combination of approaches. First, since ctDNA concentrations were almost always below 50%, putative SNPs (which should be present at MAFs of ∼50 and ∼100%) were removed using MAF-based filtering. Second, we removed SNPs previously catalogued by the Exome Aggregation Consortium (ExAC)^59^. Third, we limited our variant calls to nonsynonymous nucleotide substitutions. Lastly, we hypothesized that aberrations in genes that drive resistance should be positively selected for over the course of therapy (see ‘Resistance mechanism analysis' section below). Therefore, we only identified alterations as potential mediators of resistance if they were absent before treatment but emerged at progression or they increased in relative abundance over the course of therapy. In addition to more conservatively identifying resistance mechanisms, this approach helps to eliminate residual SNPs not present in the ExAC database since these would not be expected to change in abundance over the course of treatment. Plasma extraction and quantification statistics, as well as the mutational profiles for all the plasma samples analysed in this study, are provided as Supplementary Data 1.

### Ratio of T790M to *EGFR*-activating mutation analyses

The ratio of T790M to *EGFR*-activating mutation was calculated using the % variant allele frequency (VAF) of each mutation. In samples in which a significant copy-number gain was detected in *EGFR*, the % *EGFR*-activating mutation was corrected for the level of copy-number gain.

First, we compared the ratio of T790M to activating mutation in pre-treatment and progression plasma samples (Fig. 1c,d). All patients in whom T790M was detectable pre-treatment and activating mutations were present at both time points were considered (*n*=35 patients). Patients CO47 and CO8 were excluded from this analysis due to a low *EGFR*-activating mutation VAF post-treatment (0.06 and 0.2%) that did not allow for an accurate calculation of the change in this ratio. In patients in whom activating *EGFR* mutations were detected in progression plasma samples but T790M was not detected (*n*=7 patients), the VAF of T790M used for this analysis was the lower of the following two values: (1) The VAF corresponding to the 95% confidence limit for detecting T790M as a single variant based on the depth of sequencing at the T790M locus. (2) The VAF of the *EGFR*-activating mutation (assuming the ratio to be 1.0). The Wilcoxon signed-rank test was used to test for significance when comparing the ratio of T790M to *EGFR*-activating mutation in pre-treatment and progression plasma samples (Fig. 1d).

In a second analysis considering all patients with detectable T790M mutations in pre-treatment plasma (*n*=41; Fig. 1e) we compared the maximum amount of tumour shrinkage (best response as measured using RECIST) between patients with a low ratio of T790M to activating mutation (≤0.5; *n*=12) and those with a high ratio of T790M to activating mutation (>0.5; *n*=29). A ratio of 0.5 was determined to be the optimal threshold by an ROC analysis for the likelihood of a partial response (per RECIST guidelines) based on the ratio of T790M to activating mutation in pre-treatment plasma samples. The best response in patients was also significantly different between groups when using a median cut-point (*P*<0.05). The Wilcoxon rank-sum test was used to test for significance.

### Somatic copy-number alteration analysis

To identify copy-number changes in ctDNA, we defined a copy-number index for targeted SCNA analysis in which statistically significant cutoffs were empirically determined using healthy control plasma samples (see below). When analysing patient plasma samples, if a significant copy-number gain was detected the magnitude of the copy-number gain in cfDNA was normalized by the % ctDNA of the sample to estimate the mean copy number (normalized copy number) across all cells contributing ctDNA. When copy-number gains occur in a subclone, the reported normalized copy number will therefore be lower than the level present in the subclone. *TP53* mutations, which are considered to be truncal in NSCLC^21^,^22^, were used when possible to determine the % ctDNA. This was possible for approximately half of the patients analysed for SCNA (20/43 patients). For patients who did not harbour *TP53* mutations, the % *EGFR*-activating mutation, corrected for excess coverage of *EGFR*, was used.

### Analysis of sequencing depths in healthy controls

Plasma samples were obtained from 27 healthy controls that provided informed consent and were enrolled in a study approved by the Stanford University Institutional Review Board. Cell-free DNA was extracted and sequenced using our 302 kb NSCLC selector as described above. For a given genomic region of interest in a given healthy control sample, we first normalized sequencing depth to the selector-wide autosomal median depth of sequencing (to account for sample-to-sample variability in sequencing depth), and this normalized depth was then log2 transformed (hereon ‘depth' refers to this normalized and log2 transformed depth). Using the Shapiro-Wilk normality test, we confirmed that sequencing depths across non-contiguous genomic intervals (that is, targeted regions) spanning *EGFR*, *ERBB2* and *MET* were normally distributed. Moreover, depths for a given genomic region were normally distributed across our normal cohort, permitting the use of *z*-statistics to model copy-number differences across samples.

### Copy-number index determination

To call SCNA events in *EGFR, ERBB2* and *MET* from patient plasma samples, we defined a gene-specific copy-number index *C* and corresponding threshold *t*, the latter of which was empirically determined in healthy controls to minimize the false positive rate. First, we estimated the mean (*μ*) and standard deviation (*σ*) of the depth of sequencing for each targeted region across our cohort of 27 healthy control plasma samples by assuming:


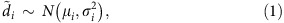


where 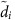 denotes the depth in region *i*. Then we calculated the corresponding Z-statistics for each region as:


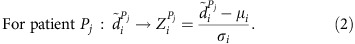


For each gene (*g*), we then integrated all measured *z*-scores (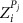) into a copy-number index (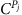), defined as the sum of gene-level *z*-scores divided by the square root of the number of targeted regions covering *g*. We note that this approach is analogous to Stouffer's method to produce an unweighted meta-*z*-score, however in this context, we are not assuming independence among the targeted regions of *g* and are therefore not computing a meta-*z*-score. Our approach requires empirical calibration in a normal cohort to derive appropriate false positive rates. A SCNA is detected if 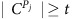 for some threshold *t*>0. For each gene of interest, we used our cohort of 27 healthy plasma samples to set *t* to the minimum value for which the false positive rate was <0.05. Copy-number index thresholds for *EGFR*, *ERBB2* and *MET* were 2.58, 3.09 and 3.045, respectively (Supplementary Fig. 6a), and copy-number indexes exceeding these thresholds were considered significant. Importantly, patient samples were normalized and analysed identically to normal controls, except for *z*-scores, which were determined using the means and standard deviations learned from the normal cohort.

### *In silico* spike to determine SCNA detection sensitivity

We performed an ‘*in silico* spike' to determine the sensitivity of detection of a 6.5 × amplification in *MET* and a 10 × amplification in *EGFR* in plasma samples with varying amounts of simulated ctDNA. For this analysis we used the cancer cell line NCI-H1573, which harbours amplifications in *MET* and *EGFR* to simulate ctDNA^60^. NCI-H1573 was ‘spiked' into healthy control cfDNA by adding sequencing reads from BAM files obtained by sequencing the NCI-H1573 cell line into BAM files generated from sequencing 27 healthy plasma samples. An adjustment was performed to account for equivalency of genomic input between samples. Twelve different spike amounts were generated for this analysis, simulating ctDNA percentages of 0.1, 0.3, 0.5, 0.75, 1, 1.25, 1.5, 1.75, 2, 2.5, 5 and 10%, generating 324 *in silico* spike samples in total. Based on this analysis, the sensitivity and specificity for detection of a 10 × amplification of *EGFR* at a copy-number index score of 2.58 are 100 and 95%, respectively, when % ctDNA is ≥2% (Supplementary Fig. 6b). Similarly, sensitivity and specificity for detection of a lower level of amplification of *MET* (6.5 ×) at a copy-number index score of 3.09 are 100 and 95%, respectively, when % ctDNA is ≥5%.

### Empirical spike

To validate the results of the *in silico* spike we performed an empirical spike experiment in which fragmented genomic DNA from NCI-H1573 cells, which harbour ∼13 copies of *MET* (6.5 × amplification) and ∼20 copies of *EGFR* (10 × amplification), was spiked into 32 ng of cfDNA from a healthy control individual at nine different concentrations ranging from 0.2 to 7%. Samples were sequenced using CAPP-Seq and SCNA analysis was performed as described above (Supplementary Fig. 6c). Congruent with the predictions from the *in silico* analyses, *EGFR* and *MET* copy-number gains were detected at concentrations as low as 0.95% and 3.37% ctDNA, respectively. In spike samples in which a significant SCNA was detected, the normalized copy number for the sample ranged from 9.4 to 9.6 and 10.1 to 15 for *MET* and *EGFR*, respectively.

### Resistance mechanism analysis

Identifying putative resistance mechanisms to rociletinib represented a unique challenge due to the fact that all the patients in this study had developed resistance to prior EGFR TKI's. Furthermore, nearly half of the patients with detectable ctDNA in pre-treatment plasma harboured one or more putative mechanisms of resistance to first- and second-generation EGFR TKI's in addition to T790M (Fig. 1b). Based on this high frequency of intra-patient heterogeneity of resistance mechanisms before rociletinib treatment, we sought to devise a method to distinguish passenger alterations and/or alterations that drive resistance to reversible EGFR TKIs (for example, T790M) from those that are driving resistance to rociletinib. Thus, we hypothesized that drivers of resistance to rociletinib would confer a competitive advantage to the clones harbouring them and would be positively selected for over the course of treatment. Therefore, in order for an alteration to be considered a putative mechanism of resistance to rociletinib (Fig. 2a–d) we required that the alteration fit one of the following two criteria: (1) the alteration was emergent (for example, absent before treatment but present at progression), or (2) the alteration increased in relative abundance over the course of therapy. For the latter criteria, the relative ratio of the alteration to the overall % ctDNA in progression plasma was required to increase compared with the ratio observed in the pre-treatment sample. *TP53* mutations, which are truncal in NSCLC^21^,^22^, were used when possible to determine the % ctDNA. This was possible for approximately half of the patients (20/43 patients). For patients who did not harbour *TP53* mutations, the % *EGFR*-activating mutation, corrected for excess coverage of *EGFR*, was used. In the scenario that a SCNA was detected pre-treatment, but SCNA analysis was inconclusive at progression due to insufficient ctDNA (<2% *EGFR*-activating mutation), the alteration was still considered a putative mechanism of resistance. In the scenario that a SNV was detected pre-treatment, but there was no detectable ctDNA at progression the alteration was still considered a putative mechanism of resistance. This was only the case for 1 SNV (*CDKN2A* D74A) in CO3.

### Innate versus acquired resistance

To assess potential patterns of resistance mechanisms, resistance to rociletinib was characterized as either innate or acquired based on the progression free survival (PFS) of the patients. Patients who progressed in <3 months were classified as having innate resistance (*n*=15), while patients with a PFS >3 months were classified as having acquired resistance (*n*=28). Next, we defined putative mechanisms of resistance to rociletinib (see prior section) as alterations that were either absent before treatment and emerged at progression or that increased in relative abundance over the course of therapy (Fig. 2a–d). Alterations conferring innate resistance had to be present in the pre-treatment plasma sample while alterations conferring acquired resistance had to be present in the progression plasma sample. Putative resistance mechanism(s) identified in each patient were categorized as SCNA only, SCNA+SNV or SNV only. The resistance mechanisms identified in patients with innate versus acquired resistance was then compared using the Freeman–Halton extension of Fisher's exact test for a two-rows by three-columns contingency table. Since there is no gold standard definition of what constitutes innate and acquired resistance, we explored a variety of different definitions, including classifications based on best response to treatment (for example, no shrinkage versus any shrinkage; >30% shrinkage versus <30% shrinkage; >10% shrinkage versus <10% shrinkage) or progression free survival (for example, ≤3 months versus >3 months; ≤4 months versus >4 months). The conclusions were identical using each categorization, with SCNAs in *MET*, *ERBB2* and *EGFR* being significantly more common in the pre-treatment plasma of patients with innate resistance, while emergent or increasing SNVs were more common in the progression plasma of patients with acquired resistance.

### L798I modelling

The template EGFR^T790M^ for modelling was PDB code 3IKA. Examination of related EGFR PDB entries indicated a highly variable C terminus region often missing from the crystal data. Specifically, 3IKA chain A with WZ4002 bound is missing LEU989-ASP1003. The PDB structure 4JIU chain B has no bound ligand but the positions of 988 and 1004 in PDB 4JIU are a good fit for the corresponding residues in 3IKA. We thus used the Prime modelling software^61^,^62^ to patch these residues from PDB 4JIU chain B into the C terminus structural gap in 3IKA. Prime was also used to repair missing side chains in 3IKA and subsequently refine the entire unified model.

The ligand is covalently bound to 3IKA but for the simulation, this covalent bond was broken to mimic the pre-reaction state. Valence state adjustments consistent with this bond breakage were also made. Then *in silico*, residue 798 was mutated from Leu to Ile. Both the EGFR^T790M^ and EGFR^T790M/L798I^ 3IKA based models were encased in an orthorhombic SPC water box with 10 Å buffer on all sides. The systems were neutralized with suitable counterions and subject to 6 nucleotides of NVT molecular dynamics at 300 K with the OPLS force field using the Desmond software package^63^,^64^.

The molecular dynamics calculations suggest that the L798I mutation affects the local orientation of nearby side chains. The Asp800 sidechain in particular is positioned in EGFR^T790M^ to H-bond to the quaternary piperazine NH+ of the ligand. This H bonding to Asp800 is completely disrupted in EGFR^T790M/L798I^. It is possible this H-bond stabilizes the ligand in the proper orientation for reactivity in EGFR^T790M^. The ligand orientation in EGFR^T790M/L798I^ is twisted in this region and this H-bond is not extant. The average distance between the ligand quaternary NH+ and the Asp800 gamma C is 2.8A in EGFR^T790M^ whereas in EGFR^T790M/L798I^ this is 6.2A. Note we measure to the gamma C as a proxy for the average position of the carboxylate oxygens which can flip in normal side chain thermal motion. The critical Cys797 sulfur to the terminal enamide distance is maintained in both EGFR^T790M^ and EGFR^T790M/L798I^ models but in EGFR^T790M/L798I^, the sidechain orientation is less than optimal for reactivity, possibly due to the loss of other stabilizing interactions. H bonding interactions with Met793 are maintained in both EGFR^T790M^ and EGFR^T790M/L798I^ models but the intra protein H bonding patterns within the vicinity of the active site are changed, again impinging on the local reactivity, including diminishing the nucleophilicity of the Cys797 sulfur.

We utilized Prime version 4.0 and Desmond Molecular Dynamics System software to perform the *in silico* modelling of the L798I mutation^61^,^63^. These software packages are available for download at http://www.schrodinger.com/downloadcenter/.

### Wild-type EGFR and ERBB2 overexpressing cells

NCI-H1975 cells were seeded in complete media at 2 × 10^5^ cells per well in a 6-well dish and allowed to adhere overnight. Next, cells were transduced at a multiplicity of infection (MOI) of ∼30 with lentiviral particles expressing the human full-length wild-type EGFR (Genecopoeia, cat. no. LPP-AO275-Lv105-050) or ERBB2 (GenTarget, cat. no. LVP504). Infections were performed in complete media for 24 h before cells were washed with PBS and fed with complete media containing 2.5 μg ml^−1^ puromycin (Life Technologies, cat. no. A11138) or 2.5 μg ml^−1^ blasticidin (Life Technologies, cat. no. A11139). Transduced cells were passaged at least three times in the presence of puromycin or blasticidin to select for EGFR- or ERBB2-overexpressing cells, respectively, before western blotting and cell proliferation assays (Supplementary Fig. 3).

### Xenograft studies

All the procedures related to animal handling, care and treatment in this manuscript were performed by either Charles River Laboratories or Crown Bioscience according to the guidelines approved by their respective Institutional Animal Care and Use Committees (IACUC) following the guidance of the Association for Assessment and Accreditation of Laboratory Animal Care (AAALAC). Blinding was not performed in the xenograft studies reported here.

PC-9 cell line xenograft studies were performed by Charles River Laboratories. Ten week old female Fox Chase SCID mice (CB-17/Icr-*Prkdc*^*scid*^/IcrIcoCrl, Charles River) were subcutaneously implanted with 5 × 10^6^ PC-9 tumour cells in 50% Matrigel (injection volume of 0.2 ml per mouse). Once tumours reached an average of 285 mm^3^, animals were heuristically sorted among treatment groups, balancing the distribution of small to large sized tumour volumes among group assignments (*n*=10 mice per group). Residual animals were subjectively placed to minimize the standard error in tumour volume between groups until the number of animals assigned to each cohort satisfied the protocol design. Animals were treated with the compounds, doses and schedules indicated (Fig. 6a,b). Changes in tumour volumes were monitored twice weekly by caliper measurements. Animals were weighed daily on day 1–5, then twice weekly using a digital balance.

LU0858 xenograft studies were performed by Crown Bioscience. Tumour fragments from LU0858 stock mice were harvested at passage 10 and one tumour fragment (2–3 mm in diameter) was implanted subcutaneously at the right flank into female BALB/c mice (HuaFukang Laboratory Animal Company) that were 6–7 weeks of age at the start of dosing. When the average tumour size reached 165 mm^3^, mice were grouped (*n*=10 mice per group) using a randomized block design. Animals were treated with the compounds, doses and schedules indicated (Fig. 8). Changes in tumour volumes and body weights were monitored twice weekly by caliper measurements and a digital balance, respectively.

### Mutation and copy-number analysis for PC-9 xenograft study

Genomic DNA was extracted from vehicle treated, erlotinib resistant (ER) and rociletinib-resistant (RR) tumours collected at endpoint using the PureLink Genomic DNA Mini Kit per the manufacturer's instructions (Life Technologies). DNA extracted from NCI-H1975/PC-9 cells was used as a control for all mutation and copy-number analyses. DNA was quantified using the Quantidex DNA assay (adapted from Sah *et al*.^65^). Sequencing was performed using CAPP-Seq as detailed above as well as using the SuraSeq 500 panel (Asuragen), which detects variants in the following genes: *ABL1, AKT1, AKT2, BRAF, EGFR, FGFR1, FGFR3, FLT3, HRAS, KIT, KRAS, MET, NRAS, PDGFRA, PIK3CA* and *RET*. For the Asuragen panel, a 2-step PCR-based target enrichment was conducted using SuraSeq NGS reagents^66^. CNV calling was performed by performing a within-sample normalization for coverage depth, followed by a within-amplicon normalization across all samples to account for amplicon-specific PCR efficiencies. Gene-level amplification was computed from the average normalized coverage for all amplicons covering a particular gene, and the gene-level amplification was compared to the baseline normalized coverage to call CNVs. CNVs were called for any sample with a normalized gene ratio greater than 1.8.

### Cell culture

NCI-H1975 cells were obtained from ATCC. PC-9 cells were a kind gift from Dr. F. Koizumi (National Cancer Center Research Institute and Shien-Lab, Tokyo, Japan). Both cell lines have been authenticated by short tandem repeat profiling (Genetica) and tested for mycoplasma contamination. ER and RR cells were derived from erlotinib resistant and rociletinib-resistant PC-9 tumours collected at endpoint, respectively. All cells were maintained in RPMI-1640 (Life Technologies) supplemented with 10% fetal bovine serum (FBS; Corning), 1 × GlutaMAX (Life Technologies), and 1 × penicillin–streptomycin (Mediatech) and propagated as monolayer cultures at 37°C in a humidified 5% CO_2_ incubator.

### Generation of tumour-derived cell lines

Viable tumour fragments from ER and RR tumours were dissociated at 37 °C in a humidified 5% CO_2_ incubator using RPMI-1640 supplemented with 1 × GlutaMAX, 1 × penicillin–streptomycin, 100 U ml^−1^ hyaluronidase and 300 U ml^−1^ collagenase IV (Worthington Biochemicals). Dissociated tumours were filtered through a 40 μM nylon cell strainer (BD Falcon), pelleted, and maintained in RPMI-1640 (Life Technologies) supplemented with 10% FBS, 1 × GlutaMAX, and 1 × penicillin–streptomycin.

### Cell proliferation assays

Cells were seeded at 3,000 cells per well in RPMI-1640 supplemented with 5% FBS, 1 × GlutaMAX, and 1% penicillin–streptomycin, allowed to adhere overnight, and treated with a dilution series of test compounds for 72 h. Cell viability was determined by CellTiter-Glo (Promega), and results were represented as relative light units normalized to a dimethyl sulfoxide (DMSO)-treated control. Growth inhibition (GI_50_) values were determined by GraphPad Prism 5.04 (GraphPad Software). Erlotinib and crizotinib were obtained from Selleck Chemical.

### Western blotting and phospho-RTK arrays

Annotated complete scans of all the western blots depicted in the manuscript are shown in Supplementary Fig. 7. PC-9, ER and RR cells were seeded at 2 × 10^6^ cells per 10 cm^2^ dish in RPMI-1640, 10% FBS, 1 × GlutaMAX and 1% penicillin–streptomycin and allowed to adhere overnight. Cells were treated with the indicated compounds for 1 h and collected in lysis buffer containing 1 × phenylmethanesulfonyl fluoride (Sigma), 1 × cell extraction buffer (Life Technologies), 1 × protease inhibitor cocktail (Enzo Life Sciences), 1 × phosphatase inhibitor cocktails I and II (EMD Chemicals). Total protein concentration was determined using a standard Bradford assay and measured on a NanoDrop 2000 spectrophotometer (Thermo Scientific). Western blotting was performed on cell lysates normalized to 30 μg total protein in loading buffer (LI-COR). Normalized lysates were run on SDS-PAGE and transferred to a nitrocellulose membrane (Life Technologies). The membrane was incubated in Qentix signal enhancement solution (Thermo Scientific), blocked with Odyssey Blocking Buffer (LI-COR, cat. no. 926-40000), and incubated overnight at 4 °C with primary antibodies. All of the primary antibodies were obtained from Cell Signaling Technology and used at a final dilution of 1:1,000 in a 1:1 solution of Odyssey Blocking Buffer and wash buffer (Phosphate Buffered Saline + 0.1% Tween-20). The primary antibodies used for this study were: total EGFR (cat. no. 2239), p-EGFR (Y1068, cat. no. 3777), total MET (cat. no. 3127), p-MET (Y1234/1235, cat. no. 3077), total AKT (cat. no. 2967), p-AKT (S473, cat. no. 9271), total MAPK (cat. no. 9107), p-MAPK (T202/Y204, cat. no. 4370), total ERBB2 (cat. no. 2248), p-ERBB2 (Y1248, cat. no. 2247), α/β-Tubulin (cat. no. 2148), and α-Tubulin (cat. no. 3873). Membranes were washed, incubated with IRDye secondary antibodies (LI-COR, cat. no. 926-68020 (goat anti-mouse) and 926-32211 (goat anti-rabbit)), washed again, and imaged on an Odyssey Fc (LI-COR). Secondary antibodies were used at a final dilution of 1:20,000. To ensure clear separation of bands for all western blots, aliquots of normalized samples were run in duplicate and blots were sectioned into strips. The higher molecular weight sections were used to detect total and phospho EGFR, ERBB2 or MET. The lower molecular weight sections were used to detect total and phospho AKT and MAPK, or tubulin. Phospho-RTK Arrays (R&D Systems) were used to assess the receptor tyrosine kinase phosphorylation status of vehicle treated and RR tumour lysates (200 μg each) per the manufacturer's instructions.

### MET shRNA knockdown experiments

PC-9 parental and RR cells were seeded in complete media at 2 × 10^5^ cells per well in a 6-well dish and allowed to adhere overnight. Next, cells were transduced at a MOI of 0.5 or 1.5 with lentiviral particles expressing a non-targeting scrambled control shRNA (Santa Cruz Biotechnology, cat. no. sc-108080), or a pool of three c-Met-specific shRNAs (Santa Cruz Biotechnology, cat. no. sc-29397-V), respectively. Infections were performed in complete media supplemented with 8 μg ml^−1^ polybrene (Santa Cruz Biotechnology, cat. no. sc-134220) for 24 h, washed with PBS, and fed with full media containing 2.5 μg ml^−1^ puromycin (Life Technologies, cat. no. A11138). Transduced cells were passaged at least three times in the presence of 2.5 μg ml^−1^ puromycin to select for shRNA-expressing cells before western blotting and cell proliferation assays that were performed as previously described.

### Statistical analyses

The statistical tests used for each experiment are listed in the main text and figure legends. Tests included the unpaired two-sided student t-test, Wilcoxon's rank-sum test, Wilcoxon signed-rank test, and the log-rank test. Variances between groups were checked for similarity and significance was defined based on *P*<0.05. No statistical method was used to predetermine sample sizes.

### Data availability

Sequence data including the somatic copy-number alteration spike experiment have been deposited into the NCBI Sequence Read Archive (SRA) under accession code SRP073668. All other relevant data are available from the authors.

## Additional information

**Accession codes:** The sequence data including the somatic copy-number alteration spike experiment have been deposited in the NCBI Sequence Read Archive (SRA) under accession code SRP073668.

**How to cite this article:** Chabon, J. J. *et al*. Circulating tumour DNA profiling reveals heterogeneity of EGFR inhibitor resistance mechanisms in lung cancer patients. *Nat. Commun.* 7:11815 doi: 10.1038/ncomms11815 (2016).

## Supplementary Material

Supplementary Supplementary Figures 1-7 and Supplementary Tables 1-2.

Supplementary Data 1 CAPP-Seq mutational profiles of the 43 rociletinib treated patients.
