# Corrigendum: Circulating tumour DNA profiling reveals heterogeneity of EGFR inhibitor resistance mechanisms in lung cancer patients

**DOI:** 10.1038/ncomms13513

**Published:** 2016-11-14

**Authors:** Jacob J. Chabon, Andrew D. Simmons, Alexander F. Lovejoy, Mohammad S. Esfahani, Aaron M. Newman, Henry J. Haringsma, David M. Kurtz, Henning Stehr, Florian Scherer, Chris A. Karlovich, Thomas C. Harding, Kathleen A. Durkin, Gregory A. Otterson, W. Thomas Purcell, D. Ross Camidge, Jonathan W. Goldman, Lecia V. Sequist, Zofia Piotrowska, Heather A. Wakelee, Joel W. Neal, Ash A. Alizadeh, Maximilian Diehn

*Nature Communications* 7: Article number: 11815 ; DOI: 10.1038/ncomms11815 (2016); Published 06 10 2016; Updated 11 14 2016

Previous work by Del Re *et al.* describing the emergence of KRAS mutations following treatment of non-small cell lung cancer patients with EGFR tyrosine kinase inhibitors was inadvertently omitted from the reference list of this Article and should have been cited as follows. The statement in the Results section ‘While it is well established that KRAS activation is a mechanism of acquired resistance in colorectal cancer patients treated with EGFR-targeting monoclonal antibodies (mAbs)^25,26,31,32^, this is to our knowledge the first report of EGFR mutant NSCLC patients acquiring activating mutations in KRAS following treatment with an EGFR TKI^10,11,23'^, and the identical statement in the Discussion section, should both have read ‘While it is well established that KRAS activation is a mechanism of acquired resistance in colorectal cancer patients treated with EGFR-targeting monoclonal antibodies (mAbs)^25,26,31,32^, here we show that EGFR mutant NSCLC patients can also acquire activating mutations in KRAS following treatment with a third generation EGFR TKI. The acquisition of KRAS mutations in EGFR mutant NSCLC patients following treatment with first line EGFR TKIs has recently been reported (Del Re *et al.*), although these mutations have not been detected in other similar first line cohorts^10,11,23,24'^.

Del Re *et al.* contribution of KRAS mutations and c.2369C>T (p.T790M) EGFR to acquired resistance to EGFR-TKIs in EGFR mutant NSCLC: a study on circulating tumor DNA. *Oncotarget* doi: 10.18632/oncotarget.6957 (2016)

