# Supplementary material for: Circulating tumour DNA profiling reveals heterogeneity of EGFR inhibitor resistance mechanisms in lung cancer patients
Source: Nat Commun. 2016 Jun 10;7:11815. doi: 10.1038/ncomms11815 (PMC4906406; doi:10.1038/ncomms11815)
Supplement: Supplementary — Figures 1-7 and Supplementary Tables 1-2. [file ncomms11815-s1.pdf]

a

Patient ID	Baseline Genomic Alterations	Baseline Alterations Present at Progression	Emergent Alterations
CO3	*CDKN2A* D74A	None	
CO4	*EGFR* only	*EGFR* only	*CDKN2A* D74A
CO7	*EGFR* only	*EGFR* only	*PIK3CA* E542K<br>*PIK3CA* E545K
CO9	*EGFR* only	*EGFR* only	*ALK* R1061Q
CO10	*EGFR* S768I	*EGFR* S768I	
CO11	*EGFR* only	*EGFR* only	*CDKN2A* D74A
CO15[#]	*RB1* G587*	*RB1* G587*	
CO16			*RB1* R787Q
CO19	*PIK3CA* N345K	*PIK3CA* N345K	*KRAS* G12A
CO22	*EGFR* G724S	*EGFR* G724S	
CO24	*RB1* S485C<br>*PIK3CA* E545K	None	
CO27	*EGFR* A750P	*EGFR* A750P	
CO29	*RB1* V654M<br>*PIK3CA* C420R<br>*PIK3CA* E545K	*RB1* V654M<br>*PIK3CA* E545K	*PIK3CA* E542K
CO31	*RB1* R251*<br>*PIK3CA* N345S	*RB1* R251*<br>*PIK3CA* N345S	*PIK3CA* E545K
CO33	*EGFR* only	*EGFR* only	*PIK3CA* E542K<br>*PIK3CA* E545K
CO34	*EGFR* only	*EGFR* only	*EGFR* L798I
CO37	*EGFR* only	*EGFR* only	*EGFR* L692V
CO39	*RB1* Y606*	*RB1* Y606*	
CO43	*EGFR* only	*EGFR* only	*EGFR* C797S
CO44	*EGFR* only	*EGFR* only	*KRAS* A146T<br>*KIT* L576P
CO45	*MET* D1304H	*MET* D1304H	*KRAS* Q61H<br>*PIK3CA* E81K
CO46	*PTEN* K125T	*PTEN* K125T	*EGFR* E709K

b

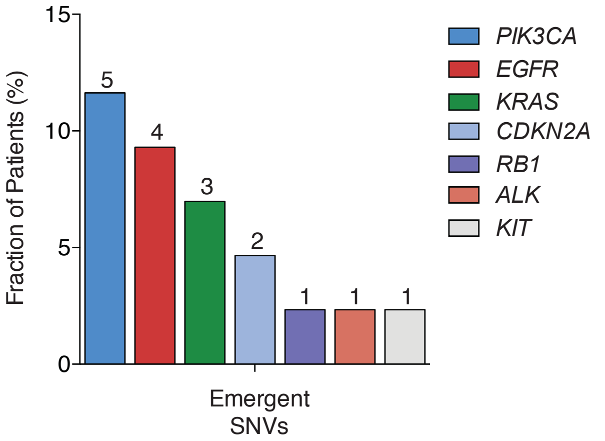

c

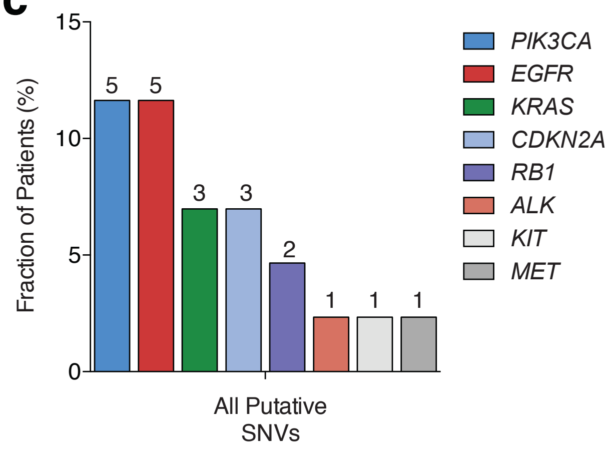

Supplementary Figure 1. Summary of Baseline and emergent SNV's in rociletinib treated patients.
(**a**) Baseline and emergent single nucleotide variants (SNVs) detected by CAPP-Seq in the plasma of patients treated with rociletinib. Only patients in whom non-*EGFR* activating and T790M mutations were detected are listed and mutations in *TP53* are excluded (* indicates baseline mutations were observed in a pre-treatment tissue biopsy but not plasma due to undetectable levels of ctDNA). (**b**) The fraction of patients in whom an emergent (absent at baseline and detected following treatment) SNV was detected in the genes listed. (**c**) The fraction of patients in whom any SNV was identified as a putative mechanism of resistance in the genes listed. This includes emergent SNVs as well as SNVs that were present at baseline and increased in relative abundance following treatment with rociletinib.

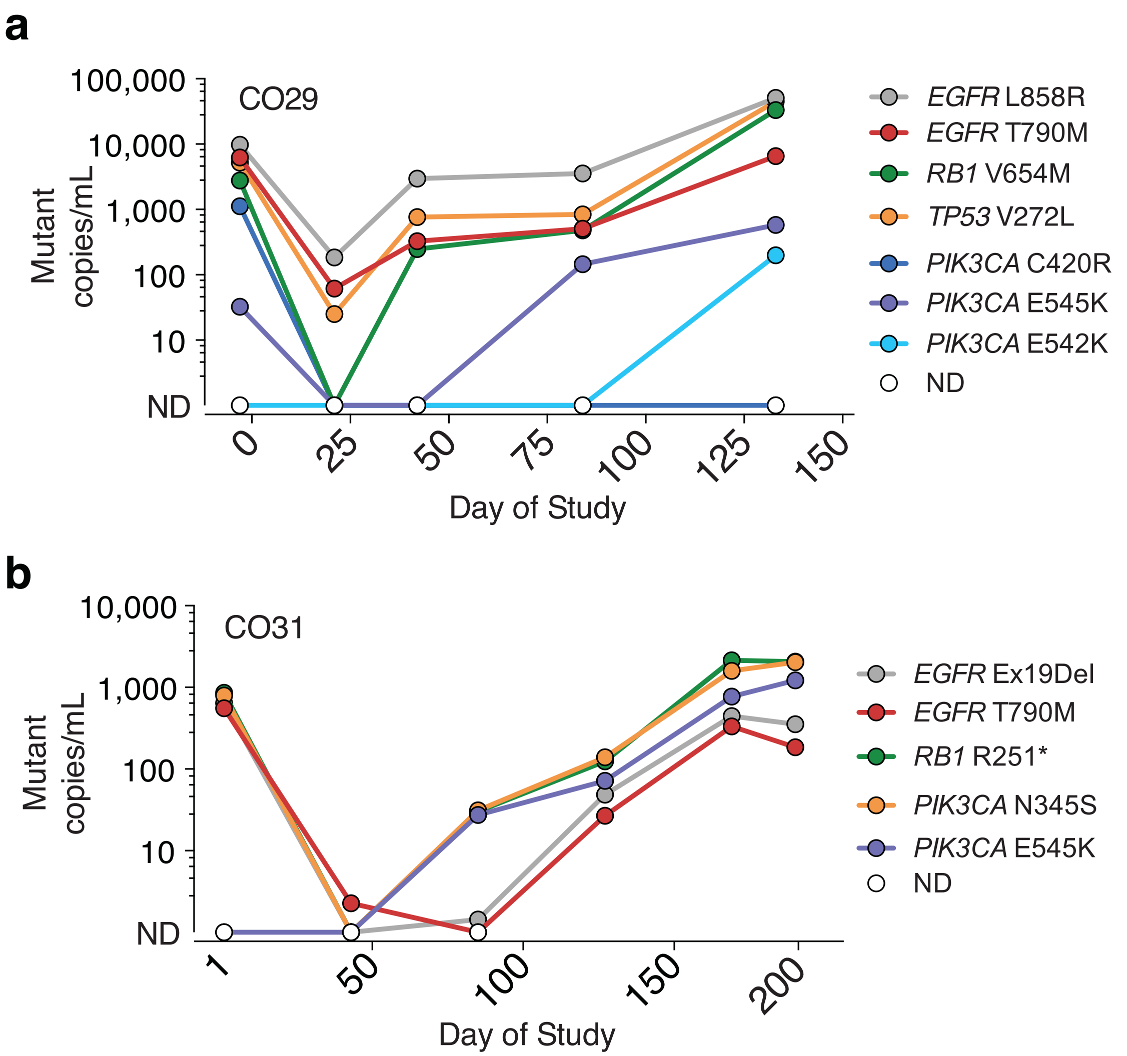

Supplementary Figure 2. Vignettes of patients with emergent *PIK3CA* mutations.
(**a-b**) Representative vignettes of patients with emergent *PIK3CA* mutations. Baseline and emergent SNVs detected by CAPP-Seq in the plasma of patients treated with rociletinib are displayed. ND="not detected".

a

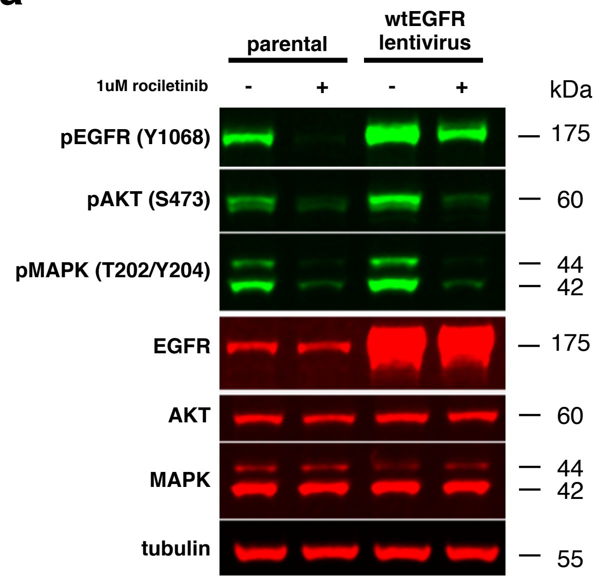

b

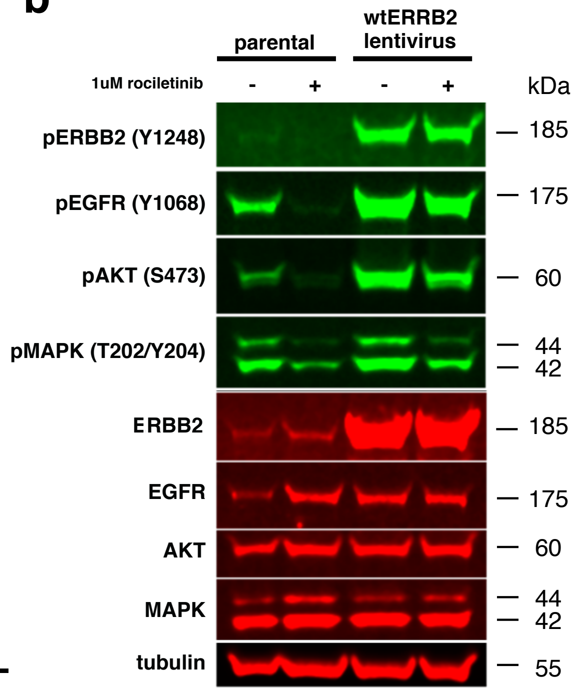

c

Treatment	NCI-H1975	NCI-H1975 wtEGFR	NCI-H1975 wtHER2
rociletinib	47 ± 26	102 ± 33*	176 ± 57***
fold over parental	NA	2.2	3.7

Supplementary Figure 3. Wild-type EGFR and ERBB2 overexpression decreases rociletinib sensitivity.
The NCI-H1975 (L858R/T790M EGFR) NSCLC cell line was transduced with lentiviral vectors expressing full-length wild-type (wt) EGFR or ERBB2, and cell populations were selected for infected cells using puromycin and blasticidin, respectively. The expression of EGFR (**a**) and ERBB2 (**b**) and markers of downstream signaling were evaluated by Western blot analysis in the presence and absence of 1μM rociletinib for 1 hour in full media. (**c**) Cell viability was evaluated in rociletinib treated parental, EGFR, and ERBB2 overexpressing cell lines after 72 hrs of treatment. Experiments were performed in triplicate, repeated three times, and data are reported as the mean percentage viability ± SD relative to control. Both EGFR and ERBB2 overexpression significantly reduced the potency of rociletinib, by 2.2- and 3.7-fold, respectively (**P* < 0.05 for EGFR, ****P* < 0.0005 for ERBB2, Wilcoxon rank-sum test).

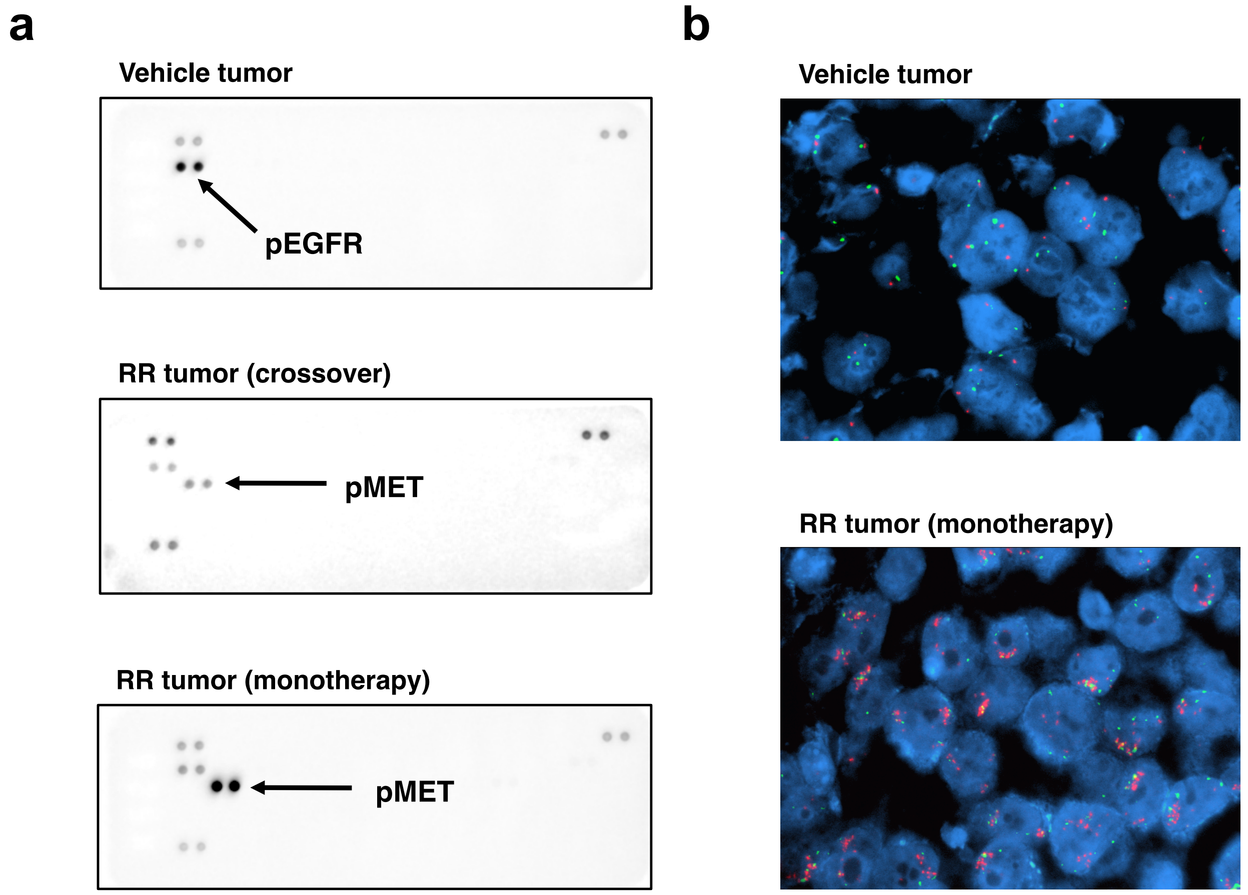

Supplementary Figure 4. *MET* amplification and pathway activation is associated with acquired resistance to rociletinib.
(**a**) Tumors were collected at endpoint from vehicle and rociletinib resistant (RR) treated animals. Tumor lysates were prepared and exposed to receptor tyrosine kinase (RTK) profiler arrays, which simultaneously detect 42 different phosphorylated RTKs. The spots not labeled with an arrow in at least one array are reference spots used to align the template. (**b**) Fluorescence in-situ hybridization (FISH) analysis was performed on vehicle and RR tumors. The *MET*/CEP7 ratio for vehicle and RR tumors are 1.98 and 5.79, respectively. *MET* staining is in red and CEP7 staining is in green. Images were taken at a magnification of 100X.

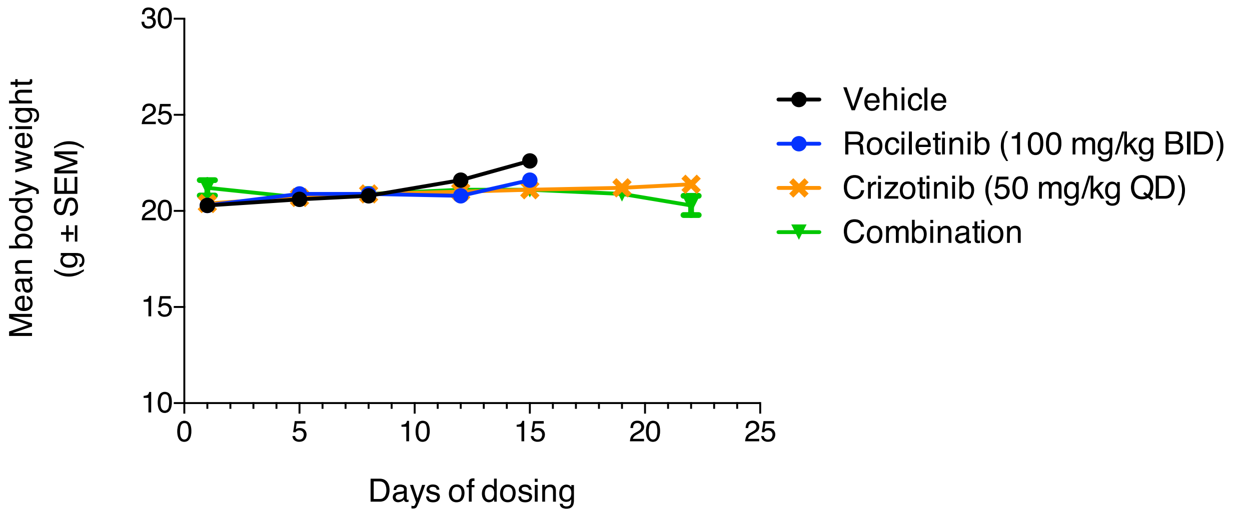

Supplementary Figure 5. Combined treatment with rociletinib and crizotinib does not lead to weight loss in mice.
LU0858 patient-derived xenograft-bearing animals were orally administered rociletinib, crizotinib, or the combination using the dose and schedules indicated (*n* = 10 mice/group) and body weights were measured twice weekly (the average weight of the mice is plotted and error bars represent ± standard error).

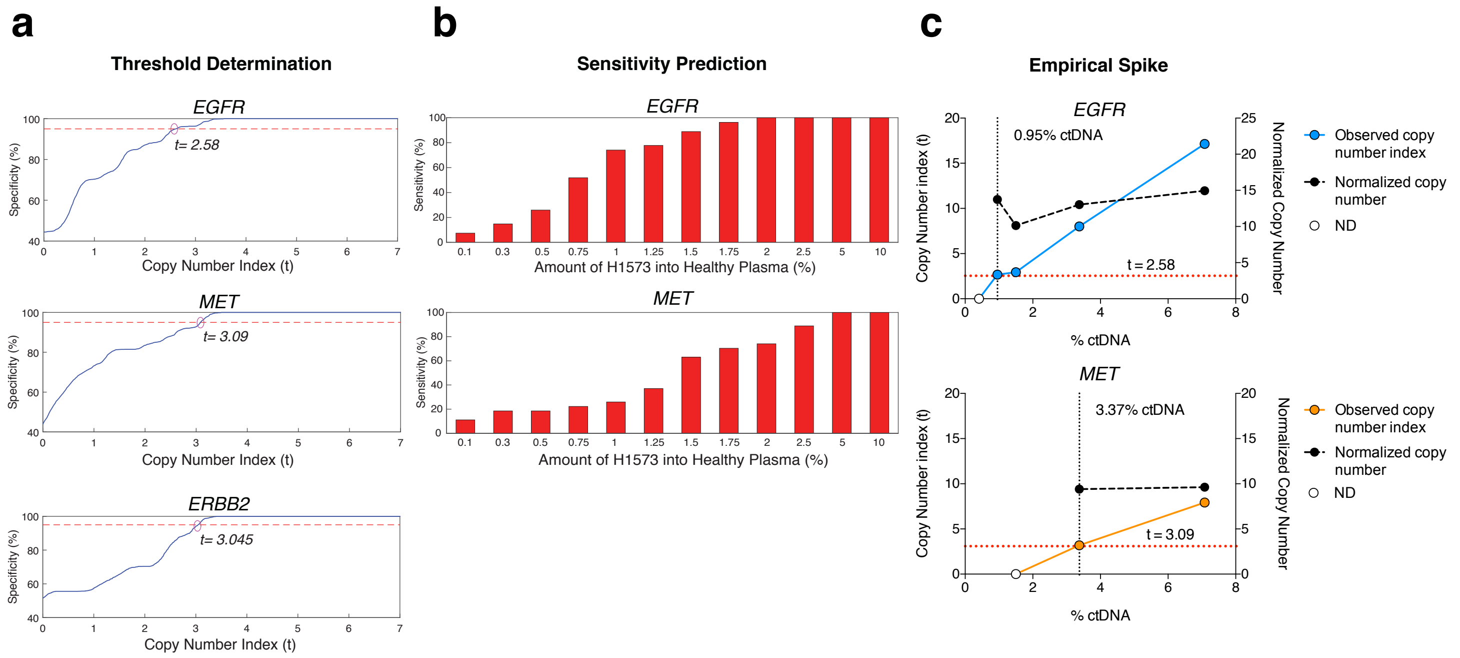

Supplementary Figure 6. Copy number index threshold determination and validation for somatic copy number alteration assessment.

(**a**) Copy number index threshold (t) determination for *EGFR*, *MET*, and *ERBB2*. The threshold (t) at which a copy number gain was considered significant in the plasma was defined as the copy number index value at which 95% specificity was achieved when analyzing 27 healthy control plasma samples. (**b**) *In-silico* spiking analysis to predict the sensitivity of detection for *EGFR* and *MET* copy number gains*.* Sequencing reads from the NCI-H1573 cancer cell line, which harbors ~20 copies of *EGFR* and ~13 copies of *MET*, were "spiked" into sequencing data from the cfDNA of 27 healthy individuals at varying ratios (0.1-10%). (**c**) Empirical spike experiment to determine the sensitivity of somatic copy number alteration (SCNA) detection in plasma. Fragmented genomic DNA from NCI-H1573 cells was spiked into cfDNA from a healthy control individual at 9 different concentrations ranging from 0.2-7%. Samples were sequenced using CAPP-Seq and SCNA analysis was performed. Copy number index thresholds (t) were determined in (**a**) and are indicated with a horizontal red dashed line. *EGFR* and *MET* copy number gains were detected at concentrations as low as 0.95% and 3.37% ctDNA, respectively (vertical black dashed line), congruent with the predictions from (**b**). The normalized copy number in samples in which a SCNA was detected ranged from 9.4-9.6 and 10.1-15 for *MET* and *EGFR*, respectively.

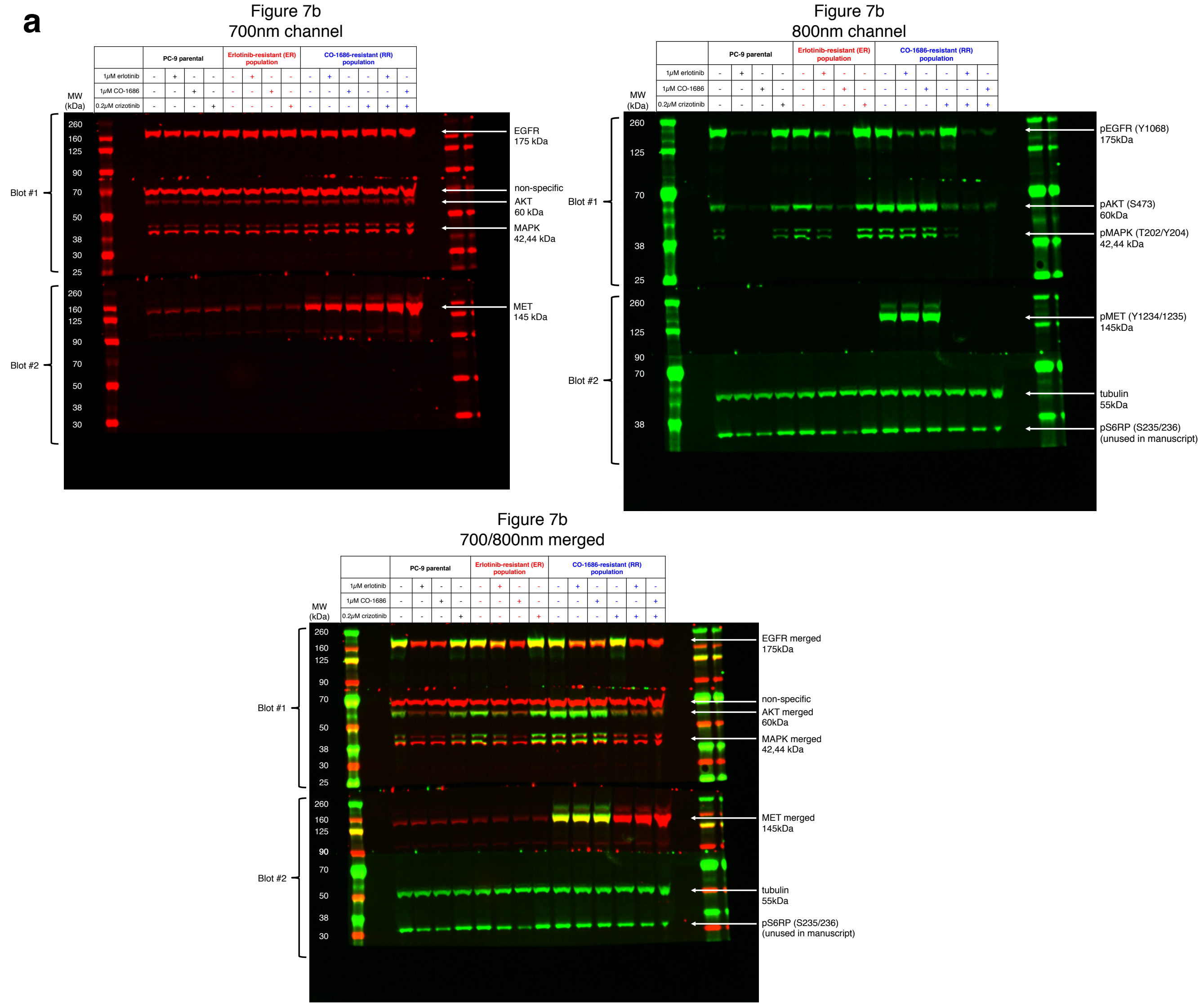

Supplementary Figure 7. Complete scans of western blots depicted in the manuscript. (**a**) Annotated raw and merged scans of the western blot depicted in **Figure 7b**.

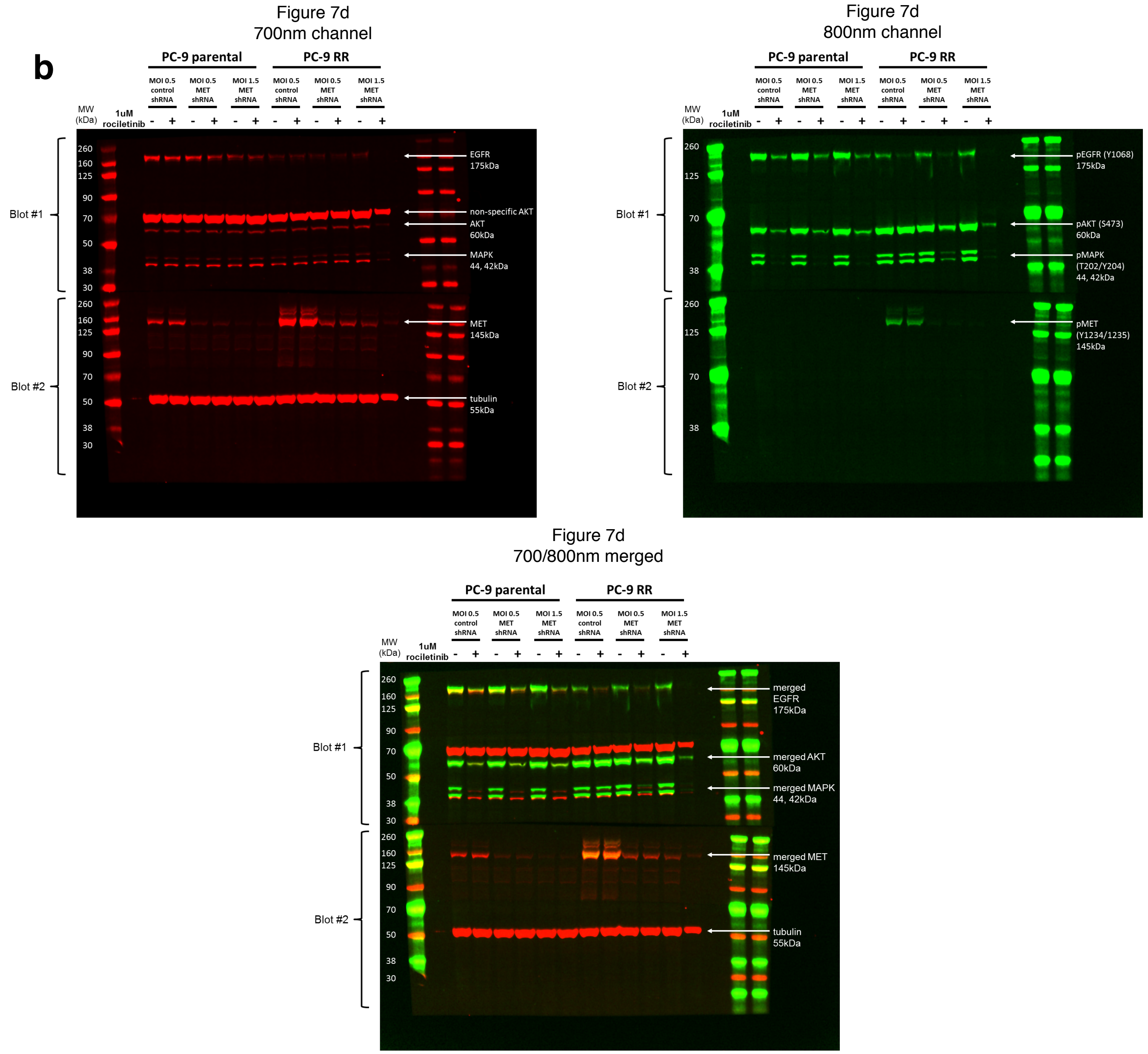

Supplementary Figure 7 continued. Complete scans of western blots depicted in the manuscript.

(**b**) Annotated raw and merged scans of the western blot depicted in **Figure 7d**. In these blots PC-9 parental and PC-9 RR cells were infected at a MOI of 0.5 or 1.5 as indicated with lentivirus harboring constructs to overexpress shRNA targeting MET or a scrambled control sequence. The results were comparable using both concentrations of lentivirus, and **Figure 7d** only shows the results from the infections at a MOI of 0.5.

c

Supplementary Figure 3a
700nm channel

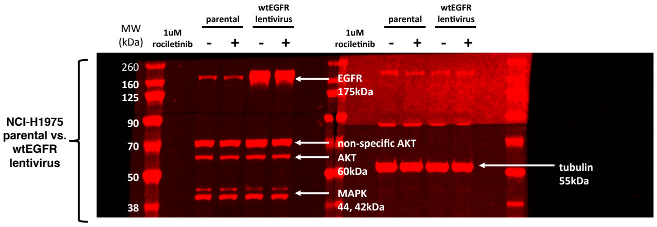

Supplementary Figure 3a
800nm channel

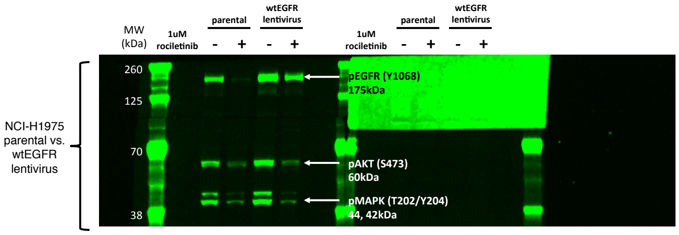

Supplementary Figure 3a
700/800nm merged

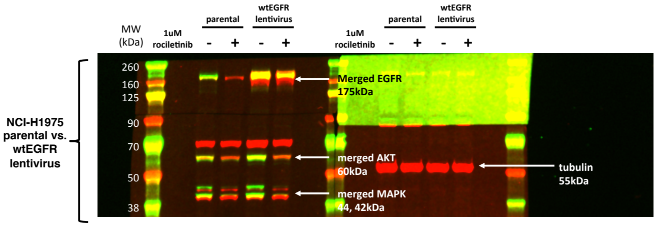

Supplementary Figure 7 continued. Complete scans of western blots depicted in the manuscript.
(**c**) Annotated raw and merged scans of the western blot depicted in **Supplementary Figure 3a**. Blots were sectioned into strips for incubation with different primary antibodies before being reassembled prior to imaging. The background intensity is different in some strips based on the primary antibodies used.

d

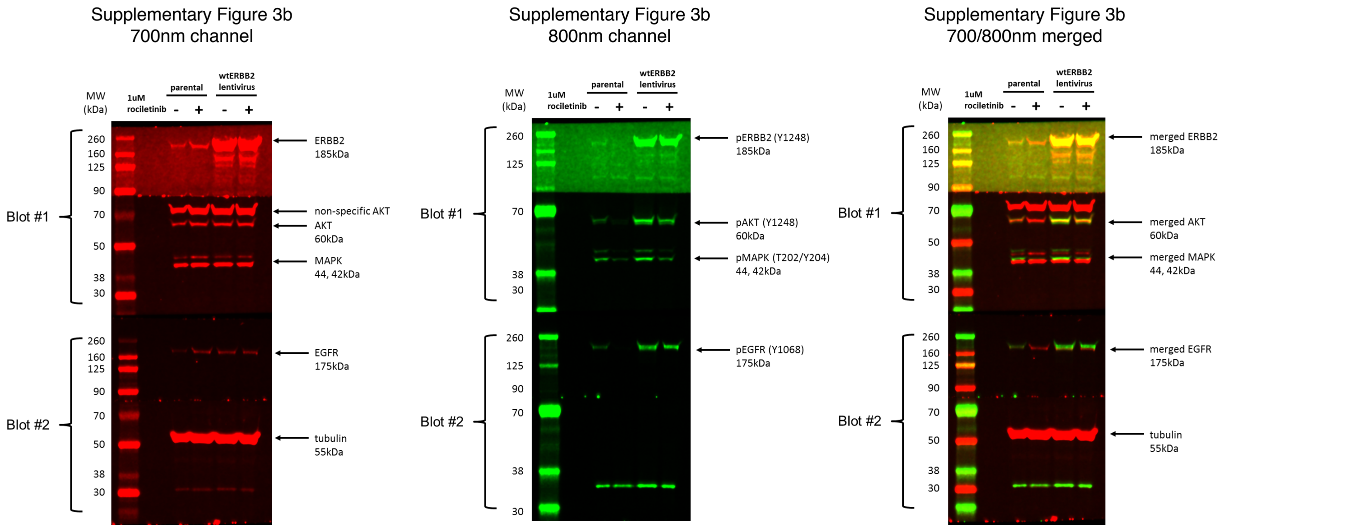

Supplementary Figure 7 continued. Complete scans of western blots depicted in the manuscript.
(**d**) Annotated raw and merged scans of the western blot depicted in **Supplementary Figure 3b**. Blots were sectioned into strips for incubation with different primary antibodies before being reassembled prior to imaging. The background intensity is different in some strips based on the primary antibodies used. Unrelated western blots scanned in parallel were cropped from the left and right of each panel shown.

Supplementary Table 1. Characteristics of the 43 rociletinib-treated patients profiled by CAPP-Seq

Patient ID	Smoking Status	Gender	Age	Race	Immediately Prior EGFR TKI	Prior History of CNS Disease	Prior Lines of Therapy (EGFR Directed)	Baseline ECOG Status	Starting Dose	Best Response by Investigator (%)	PFS by Investigator (month)	Duration on Rociletinib (month)	Duration on Rociletinib after Progression (month)[a]
CO3	Never Smoked	F	53	WHITE	NA	N	4(1)	1	900 mg BID FB	-56	4.6	15.1	10.5
CO4	Former Smoker	M	71	WHITE	erlotinib	N	6(3)	1	900 mg BID FB	32	1	1.2	
CO5	Never Smoked	F	53	WHITE	erlotinib	Y	5(4)	1	900 mg BID FB	-10	1.4	6.2	4.8
CO6	Never Smoked	F	51	WHITE	erlotinib	Y	7(7)	1	900 mg BID FB	-31	4	5.5	1.5
CO7	Never Smoked	M	48	WHITE	erlotinib	Y	3(1)	1	500 mg BID HBr	26	1.3	2.1	0.8
CO8	Never Smoked	F	53	WHITE	erlotinib	N	1(1)	0	500 mg BID HBr	41	1	1.2	
CO9	Never Smoked	M	74	WHITE	afatinib	N	4(2)	1	1000 mg BID HBr	30	1.3	1.4	
CO10	Never Smoked	F	55	WHITE	erlotinib	N	3(1)	0	500 mg BID HBr	-32	3.9	7.3	3.4
CO11	Never Smoked	F	30	WHITE	NA	N	4(1)	1	750 mg BID HBr	-71	2.7	6.4	3.7
CO14	Never Smoked	M	64	WHITE	NA	Y	2(1)	0	400 mg TID FB	-24	4	11.4	7.4
CO15	Never Smoked	F	48	ASIAN	erlotinib	N	4(1)	1	900 mg BID FB	-71	12.2	13.3	1.1
CO16	Never Smoked	F	48	ASIAN	erlotinib	Y	6(1)	1	625 mg BID HBr	8	2.8	2.8	
CO17	Never Smoked	F	64	WHITE	erlotinib	Y	5(2)	1	750 mg BID HBr	-58	2.9	2.9	
CO18	Never Smoked	M	53	WHITE	NA	N	5(2)	0	200 mg QD FB	-22	13.5	14.9	1.4
CO19	Never Smoked	F	66	ASIAN	NA	Y	2(1)	1	750 mg BID HBr	-41	4.1	4.6	0.5
CO20	Never Smoked	F	60	WHITE	NA	Y	4(2)	1	625 mg BID HBr	8	1.3	2.5	1.2
CO21	Never Smoked	M	58	WHITE	afatinib	N	3(2)	1	625 mg BID HBr	15	5	0.7	
CO22	Never Smoked	F	55	WHITE	erlotinib	Y	3(2)	0	625 mg BID HBr	0	1.1	1.1	
CO23	Never Smoked	F	62	WHITE	erlotinib	Y	5(2)	1	750 mg BID HBr	-5	3.8	12.4	8.6
CO24	Never Smoked	F	57	WHITE	NA	Y	2(1)	0	625 mg BID HBr	-47	2.8	5.8	3
CO25	Never Smoked	F	47	WHITE	erlotinib	N	6(1)	1	500 mg BID HBr	-42	6	8.8	2.8
CO26	Never Smoked	M	47	WHITE	erlotinib	Y	7(2)	1	750 mg BID HBr	-39	6.1	6.1	
CO27	Never Smoked	F	67	ASIAN	erlotinib	Y	6(3)	0	750 mg BID HBr	-14	4.2	4.2	
CO28	Never Smoked	F	47	WHITE	afatinib	Y	5(2)	1	625 mg BID HBr	-67	2.6	7	4.4
CO29	Former Smoker	F	72	WHITE	afatinib	N	6(3)	1	500 mg BID HBr	-47	3.9	4.3	
CO30	Never Smoked	F	37	NOT PROVIDED	NA	Y	1(1)	1	750 mg BID HBr	-47	1.4	3.5	2.1
CO31	Never Smoked	F	84	WHITE	erlotinib	Y	1(1)	1	750 mg BID HBr	-53	6.1	5.5	
CO32	Never Smoked	M	40	NOT PROVIDED	erlotinib	N	1(1)	1	625 mg BID HBr	-60	6.1	6.2	
CO33	Never Smoked	F	56	WHITE	erlotinib	N	6(2)	1	750 mg BID HBr	-33	8.3	11.3	3
CO34	Former Smoker	M	46	ASIAN	erlotinib	N	3(2)	0	625 mg BID HBr	-33	4.1	9.5	5.4
CO35	Former Smoker	M	66	WHITE	erlotinib	N	4(3)	1	500 mg BID HBr	-9	12.7	12.7	
CO36	Former Smoker	M	72	WHITE	erlotinib	N	2(1)	0	625 mg BID HBr	-11	2.6	5.3	2.7
CO37	Never Smoked	F	54	WHITE	erlotinib	Y	3(2)	1	750 mg BID HBr	-35	7	9.6	2.6
CO39	Never Smoked	M	52	WHITE	erlotinib	Y	1(1)	1	750 mg BID HBr	-40	4.3	8.5	4.2
CO40	Never Smoked	M	59	BLACK OR AFRICAN AMERICAN	erlotinib	N	1(1)	1	500 mg BID HBr	-27	4.2	4.9	0.7
CO41	Former Smoker	M	68	WHITE	erlotinib	N	1(1)	1	750 mg BID HBr	-28	5.6	12	6.4
CO43	Never Smoked	F	45	WHITE	NA	N	2(1)	0	625 mg BID HBr	-40	6.1	10.7	4.6
CO44	Former Smoker	F	66	WHITE	erlotinib	N	4(1)	1	625 mg BID HBr	-31	5.2	9	3.8
CO45	Never Smoked	F	65	WHITE	erlotinib	N	3(1)	1	500 mg BID HBr	-50	3.9	13.1	9.2
CO46	Never Smoked	F	81	WHITE	erlotinib	N	3(2)	1	625 mg BID HBr	-4	3.9	4.1	
CO47	Former Smoker	F	66	ASIAN	afatinib	Y	8(3)	1	500 mg BID HBr	-30	3.7	3.5	
CO48	Never Smoked	F	51	WHITE	NA	Y	2(1)	1	500 mg BID HBr	7	2.8	9.7	6.9
CO50	Never Smoked	M	65	BLACK OR AFRICAN AMERICAN	erlotinib	N	5(3)	0	750 mg BID HBr	-46	6.2	5.8	

[a] Values are only listed for patients that remained on rociletinib ≥ 0.5 months after progression

Abbreviations: M = male; F = female; NA = not applicable; ECOG = Eastern Cooperative Oncology Group.

ABCC9	CTNNB1	HDAC9	MET	PRIM2	ST6GAL2
ABL1	CTSS	HRAS	MKRN3	PSPC1	STK11
ABR	CUL3	HTR1A	MPHOSPH8	PTEN	SYT4
ADAMTS12	DCAF12L1	HTR3E	MRGPRD	PTPRD	TARS2
AKT1	DCAF12L2	IGFL3	MS4A3	PXDNL	TERT
ALK	DCAF4L2	IL6R	MYC	RAF1	TG
AMOT	DCC	IQCJ-SCHIP1	MYCL	RB1	TGFBR3
ANK2	DDR2	KCNA4	MYCN	REG3A	TLR4
APOB	DDX1	KCND2	MYD88	RET	TNN
ASB18	DENND4B	KCNJ3	MYEOV	RFX5	TNR
ASTN1	DMD	KCNT2	MYNN	RIT2	TP53
ASTN2	DUSP27	KDR	NAV3	RLF	TP63
ASXL3	EGFLAM	KEAP1	NCAM1	ROBO1	TPCN2
ATP11B	EGFR	KIF2B	NETO1	ROS1	TPTE2
BAGE5	EPHA3	KIT	NF2	RP1L1	TRDMT1
BCL11B	ERBB2	KLHL1	NFE2L2	RPS4Y1	TRIM58
BCL2	ERBB4	KLHL6	NKD2	RYR2	TRIP13
BRAF	ERICH3	KMT2C	NLRP3	S100A7	TRPC5
BRINP3	FAM135B	KMT2D	NLRP5	SALL1	TSHZ3
C14orf177	FAT3	KRAS	NOTCH1	SAMD7	TUBA3C
C22orf34	FBN2	LANCL2	NRAS	SEMA6C	U2AF1
C6orf118	FBXL7	LELP1	NT5C1A	SERPINB3	UGT3A2
C9	FBXW7	LINC00441	NTM	SETBP1	VGLL4
CA10	FGD1	LMNTD1	NUP155	SF3B1	VSTM2A
CASC11	FGF3	LOC349160	PABPC4	SFTA3	WDR7
CASC8	FGFR1	LPL	PACRG	SGCZ	WHSC1L1
CCND1	FGFR2	LPPR4	PAK7	SKP2	XIRP2
CD226	FGFR3	LRFN5	PAPPA2	SLC12A7	ZAN
CDH10	FKBP9P1	LRP1B	PARK2	SLC14A2	ZFHX4
CDH12	FLJ26245	LRRC4C	PCDH10	SLC1A3	ZFY
CDH18	FOXP1	LRRIQ3	PCDH17	SLC2A2	ZIC1
CDH7	FRYL	LRRTM4	PDGFRA	SLC45A2	ZIC4
CDH9	GABRA2	MACF1	PDGFRB	SLC6A19	ZMYM5
CDKN2A	GABRA6	MAP2K1	PDHA2	SLC8A1	ZNF236
CHEK2	GPR112	MAP2K2	PDYN	SLIT3	ZNF423
CLDN11	GPR158	MAPK1	PDZRN3	SLITRK1	ZNF521
CNTNAP2	GRID1	MARCH1	PEG3	SLITRK2	ZNF536
CNTNAP5	GRIK3	MCCC1	PHC3	SLITRK3	ZNF713
COL22A1	GRM8	MCF2L2	PIK3CA	SMAD4	ZNF804A
CSMD1	HAPLN1	MCL1	PKLR	SOX2-OT	ZNF804B
CSMD3	HAX1	MDM2	POLDIP2	SPHKAP	ZNF831
CTNNA2	HCN1	MECOM	POM121L12	SPTA1	!!

Supplementary Table 2. NSCLC-focused CAPP-Seq Selector summary.
This table lists the 252 genes that were either partially or fully covered by the 302kb CAPP-Seq selector used in this study.
